# Are C-Reactive Protein Associated Genetic Variants Associated with Serum Levels and Retinal Markers of Microvascular Pathology in Asian Populations from Singapore?

**DOI:** 10.1371/journal.pone.0067650

**Published:** 2013-07-02

**Authors:** Rajkumar Dorajoo, Ruoying Li, Mohammad Kamran Ikram, Jianjun Liu, Philippe Froguel, Jeannette Lee, Xueling Sim, Rick Twee-Hee Ong, Wan Ting Tay, Chen Peng, Terri L. Young, Alexandra I. F. Blakemore, Ching Yu Cheng, Tin Aung, Paul Mitchell, Jie Jin Wang, Caroline C. Klaver, Eric Boerwinkle, Ronald Klein, David S. Siscovick, Richard A. Jensen, Vilmundur Gudnason, Albert Vernon Smith, Yik Ying Teo, Tien Yin Wong, E-Shyong Tai, Chew-Kiat Heng, Yechiel Friedlander

**Affiliations:** 1 Genome Institute of Singapore, Agency for Science, Technology and Research, Singapore, Singapore; 2 Yong Loo Lin School of Medicine, National University of Singapore, Singapore, Singapore; 3 Singapore Eye Research Institute, Singapore National Eye Centre, Singapore, Singapore; 4 Department of Ophthalmology, Yong Loo Lin School of Medicine, National University of Singapore, Singapore, Singapore; 5 Centre for Quantitative Medicine, Office of Clinical Sciences, Duke-NUS Graduate Medical School, Singapore, Singapore; 6 Department of Epidemiology and Ophthalmology, Erasmus Medical Center, Rotterdam, The Netherlands; 7 Department of Genomics of Common Disease, School of Public Health, Imperial College London, Hammersmith Hospital, London, United Kingdom; 8 CNRS-UMR-8199, Univ Lille Nord de France, UDSL, Lille, France; 9 Saw Swee Hock School of Public Health, National University of Singapore, Singapore, Singapore; 10 Centre for Molecular Epidemiology, National University of Singapore, Singapore, Singapore; 11 Centre for Statistical Genetics, University of Michigan, Ann Arbor, Michigan, United States of America; 12 National University of Singapore Graduate School for Integrative Science and Engineering, National University of Singapore, Singapore, Singapore; 13 Duke–National University of Singapore Graduate Medical School, Singapore, Singapore; 14 Duke Centre for Human Genetics, Duke University Medical Center, Durham, North Carolina, United States of America; 15 Section of Investigative Medicine, Division of Diabetes, Endocrinology and Metabolism, Imperial College London, London, United Kingdom; 16 Centre for Vision Research, Department of Ophthalmology and Westmead Millennium Institute, University of Sydney, Sydney, Australia; 17 Centre for Eye Research Australia, University of Melbourne, Royal Victorian Eye and Ear Hospital, Melbourne, Australia; 18 Human Genetics Center and Human Genome Sequencing Center, University of Texas and Baylor College of Medicine, Houston, Texas, United States of America; 19 Department of Ophthalmology and Visual Science, University of Wisconsin, Madison, Wisconsin, United States of America; 20 Cardiovascular Health Research Unit, University of Washington, Seattle, Washington, United States of America; 21 Department of Medicine, University of Washington, Seattle, Washington, United States of America; 22 Department of Epidemiology, University of Washington, Seattle, Washington, United States of America; 23 Department of Medicine, University of Iceland, Reykjavik, Iceland; 24 Icelandic Heart Association, Kopavogur, Iceland, University of Iceland, Reykjavik, Iceland; 25 Department of Epidemiology and Public Health, National University of Singapore, Singapore, Singapore; 26 Department of Medicine, Yong Loo Lin School of Medicine, National University of Singapore, Singapore, Singapore; 27 Duke–National University of Singapore Graduate Medical School, Singapore, Singapore; 28 Department of Pediatrics, Yong Loo Lin School of Medicine, National University of Singapore, Singapore, Singapore; 29 Epidemiology Unit, Hebrew University-Hadassah School of Public Health and Community Medicine, Jerusalem, Israel; New Jersey Institute of Technology, United States of America

## Abstract

**Introduction:**

C-reactive protein (CRP) levels are associated with cardiovascular disease and systemic inflammation. We assessed whether CRP-associated loci were associated with serum CRP and retinal markers of microvascular disease, in Asian populations.

**Methods:**

Genome-wide association analysis (GWAS) for serum CRP was performed in East-Asian Chinese (N = 2,434) and Malays (N = 2,542) and South-Asian Indians (N = 2,538) from Singapore. Leveraging on GWAS data, we assessed, *in silico*, association levels among the Singaporean datasets for 22 recently identified CRP-associated loci. At loci where directional inconsistencies were observed, quantification of inter-ethnic linkage disequilibrium (LD) difference was determined. Next, we assessed association for a variant at *CRP* and retinal vessel traits [central retinal artery equivalent (CRAE) and central retinal vein equivalent (CRVE)] in a total of 24,132 subjects of East-Asian, South-Asian and European ancestry.

**Results:**

Serum CRP was associated with SNPs in/near *APOE*, *CRP*, *HNF1A* and *LEPR* (p-values ≤4.7×10^−8^) after meta-analysis of Singaporean populations. Using a candidate-SNP approach, we further replicated SNPs at 4 additional loci that had been recently identified to be associated with serum CRP (*IL6R*, *GCKR*, *IL6 and IL1F10)* (p-values ≤0.009), in the Singaporean datasets. SNPs from these 8 loci explained 4.05% of variance in serum CRP. Two SNPs (rs2847281 and rs6901250) were detected to be significant (p-value ≤0.036) but with opposite effect directions in the Singaporean populations as compared to original European studies. At these loci we did not detect significant inter-population LD differences. We further did not observe a significant association between *CRP* variant and CRVE or CRAE levels after meta-analysis of all Singaporean and European datasets (p-value >0.058).

**Conclusions:**

Common variants associated with serum CRP, first detected in primarily European studies, are also associated with CRP levels in East-Asian and South-Asian populations. We did not find a causal link between CRP and retinal measures of microvascular disease.

## Introduction

Elevated serum C-reactive protein (CRP) level is a marker for increased systemic inflammation [1] and is associated with both macrovascular [i.e stroke and coronary heart disease (CHD)] [2]–[3] and microvascular [i.e diabetic nephropathy and chronic kidney disease (CKD)] [4]–[5] disease. CRP levels show significant heritability [6]. Understanding the pathways involved in the pathogenesis of elevated serum CRP may provide crucial insights into the pathophysiology of vascular diseases. Genome-wide association studies (GWAS) have successfully identified common genetic variants at 22 different gene loci that are associated with CRP levels [7]–[10]. However, several of these variants were discovered after large-scale meta-analysis using only populations of European ancestry [7]. As there are substantial differences in mean serum CRP concentrations between ethnic groups [11], it is important to understand the relevance of these recently identified variants in influencing serum CRP levels in non-European populations.

The causal link between CRP and vascular diseases is controversial and it has been suggested that elevated serum CRP may be a consequence of inflammatory processes that subsequently lead to disease outcomes [12]. While there is some evidence for a pro-inflammatory and pro-artherogenic role for CRP [13]–[14], a recent *in vivo* study showed no suggestion of atherosclerotic plaque reduction in a *CRP* knock-out mouse model [15]. Furthermore, common genetic variants associated with serum CRP levels identified from GWAS were not associated with major macrovascular outcomes such as myocardial infarction (MI) or CHD [7]–[8]. In contrast, with respect to microvascular pathology, a recent study identified a *CRP* gene variant that was associated with CKD [16]. Nevertheless, data on the link between serum CRP and evidence of microvascular variations is lacking.

In this study, we aimed to identify common genetic variants associated with serum CRP levels among Asian populations (East-Asian Chinese and Malays and South-Asian Indian populations) from Singapore, using a genome-wide association study (GWAS) approach. Leveraging on genome-wide data, we next sought to assess, *in silico*, the relevance of recently identified variants, several of which have been only detected using European populations [7], to these Asian subjects. Finally, to clarify the role of serum CRP in the pathogenesis of vascular disease, we determined whether a genetic variant at the *CRP* gene locus was associated with changes in retinal vessel traits, which have been found to be associated with serum CRP levels, subclinical vascular pathology and clinical cardiovascular outcomes [17]–[23].

## Methods

### Ethics Statement

Each cohort used in this study secured approval from their respective institutional review boards, and all participants provided written informed consent in accordance with the Declaration of Helsinki. In addition, for the Singaporean datasets, approval was granted by the Singapore General Hospital Ethics Committee, the Singapore Eye Research Institute Ethics Committee and the National Health Group Domain Specific Review Board and the genetic analysis was approved by the National University of Singapore Institutional Review Board.

### Study Samples

To assess for association with serum CRP level, data from 3 adult population-based datasets were utilized. Singapore Prospective Study 2 (SP2) is a cross-sectional study of adult Chinese (N = 5,499), Malay (N = 1,405) and Asian-Indian (N = 1,138) samples. Detailed methodology for SP2 has been previously reported [24] and GWAS data was only available for adult Chinese samples (N = 3,066) in SP2. Singapore Malay Eye Study (SiMES) is a cross-sectional study of adult Malays (N = 3,280) [25]. GWAS data was available for 3,072 Malay samples from SiMES. Singapore Indian Eye Study (SINDI) is a cross-sectional study of adult Asian-Indians (N = 3,400) living in Singapore [26] and GWAS data was available for 2,953 subjects.

For the association between the most strongly associated *CRP* variant and retinal vessel traits, we used data available in all 3 Singaporean datasets. Subsequently, additional validation was sought, *in silico*, from meta-analysis data of European samples (N = 18,722) from the Blue Mountain Eye Study (BMES), Age Gene/Environment Susceptibility - Reykjavik Study (AGES), Atherosclerosis Risk in Communities Study (ARIC), Cardiovascular Health Study (CHS) and the Rotterdam study, that had recently performed a GWAS for retinal vessel traits as part of the Cohorts for Heart and Aging Research in Genomic Epidemiology (CHARGE) consortium [27].

### Genotyping and Imputation

Genotyping and quality control procedures for SP2, SiMES and SINDI have been previously described [28]. 3,066 Chinese adults from SP2 were genotyped using the Illumina 1Mduov3 (N = 1,016), HumanHap 610Quad (N = 1,467) and Hap550 arrays (N = 583). All SiMES Malay adults (N = 3,072) and all SINDI Asian-Indian adults (N = 2,953) were genotyped using HumanHap 610Quad arrays.

Chip-wise QC procedures were conducted in a standardised manner in all studies. Tables S1 and S2 in File S1 provide study-specific details. Briefly, samples were excluded based on sample call-rates (<95%) and extreme heterozygosities (<0.25 or >0.35) (Table S1 in File S1). Identity-by-state measures were performed by pair-wise comparison of samples to detect cryptic relatedness (Table S1 in File S1). One sample from each relationship was excluded from further analysis and where duplicate samples had been genotyped in different SNP-arrays, samples from the denser array were retained.

Population structure ascertainment to prevent confounding of results, was done using principal component analysis (PCA) [29] and analysing plots with four reference panels from International Hapmap [30] and Singaporean Chinese, Malay and Asian-Indian samples from the Singapore Genome Variation Project (SGVP) [31]. Outliers with discordant ethnic membership from reported ethnicities were subsequently detected and removed (Table S1 in File S1). Samples with discrepant genetically-inferred and reported genders were also removed (Table S1 in File S1). 2,434 SP2 samples, 2,542 SiMES samples and 2,538 SINDI samples were available for statistical analysis after QC measures.

Details of quality-control measures for SNPs are provided in Table S2 in File S1. Briefly, sex-linked and mitochondrial SNPs were removed, together with gross HWE outliers (p-value <1×10^−4^). SNPs that were monomorphic or with a MAF less than 5% and SNPs with low call-rates (<95%) were also excluded. In SP2 where more than one chip was used for genotyping, the Mantel-extension test was carried out and 62 SNPs with extreme discrepancies in allele frequencies from samples genotyped on different arrays were removed from analyses.

We imputed for additional genotypes in all datasets with IMPUTE v0.5.0. [32] using a posterior probability of at least 0.90 and a call-rate of at least 95% for all imputed SNPs. Genotype calls were based on HapMap East-Asian samples (CHB and JPT) [30] for SP2 Chinese and to better capture local patterns of haplotype variation, all four HapMap reference panels (CEU, YRI and JPT+CHB) [34] for the Malay and Asian-Indian samples. 1,745,429 SNPs from SP2, 1,559,514 SNPs from SiMES and 1,527,744 SNPs from SINDI were available for subsequent analyses after imputation and QC procedures (Table S2 in File S1).

### Main Outcome Variables

#### Serum C-reactive protein levels

Clinical measures from the Singaporean datasets used in this study are presented in Table 1. Venous blood was drawn following a 10-hour overnight fast was drawn from SP2 samples and in the non-fasted state from SiMES and SINDI. Serum CRP was measured in all sample collections from frozen plasma stored at −80°C, using a particle-enhanced immunoturbidimetric method implemented on a Roche/Integra 400 Analyser (Roche Diagnostics, Rotkreuz, Switzerland). Samples with CRP measures >10 mg/L (184 samples from SiMES, 44 samples from SP2 and 242 from SINDI) that may indicate acute clinical inflammation were excluded from subsequent analysis, as recommended by the Centers for Disease Control and Prevention and the American Heart Association [33]–[34]. Raw CRP values were normalised by log transformation and used as the dependent variable in GWAS analyses.

**Table 1:** Clinical measures in SiMES, SP2 and SINDI datasets used in study.

	SP2	SiMES	SINDI
Ethnicity	Chinese	Malay	Asian-Indian
*N*	2434	2542	2538
% Males	46.6	49.5	51.1
Mean age (SD)	48.0 (11.25)	59.1 (11.04)	58.0 (10.07)
CRP [*N* *,Mean(SD)]/mg/L	2198, 1.5 (1.62)	2275, 2.5 (1.7)	2238, 2.7 (2.35)
CRVE [*N*,Mean(SD)]/µm	1411, 218.9 (20.23)	2230, 219.2 (22.61)	2390, 208.2 (19.93)
CRAE [*N*,Mean(SD)]/µm	1413, 144.8 (14.54)	2181, 141.3 (16.64)	2390, 144.6 (14.46)
BMI [*N*,Mean(SD)]/kg/m^2^	2431, 22.9 (3.39)	2522, 26.4 (5,11)	2531, 26.2 (5.03)
LDL-C [*N*,Mean(SD)]/mmol/L	2431, 3.1 (0.83)	2541, 3.5 (1.01)	2538, 3.3 (0.94)
HDL-C [*N*,Mean(SD)]/mmol/L	2432, 1.5 (0.36)	2541, 1.4 (0.33)	2537, 1.1 (0.30)
% diabetes†	5.4	25.6	33.5
% hypertension‡	34.7	68.5	78.7
% currently smoking	9.9	34.7	14.5

* Excluding 242 samples from SINDI, 190 samples from SiMES and 48 samples from SP2, respectively who had CRP values >10 mg/L.

† hypertension defined as history of hypertension or SBP>140 mmHg or DBP>90 mmHg.

‡ diabetes defined as participants with a history of diabetes mellitus or fasting glucose levels ≥7.0 mmol/L in SP2 and HbA1c levels ≥6.5% in non-fasting blood samples and/or those with previous history in SiMES and SINDI.

#### Retinal markers of microcirculation

Central retinal vein equivalent (CRVE) and central retinal artery equivalent (CRAE) measures were similarly determined in all datasets. Digital fundus photographs were taken using a retinal camera (Canon CRDGi with a 10D SLR back; Canon, Tokyo, Japan) after pupil dilation. Two retinal images were taken, according to the Early Treatment for Diabetic Retinopathy Study (ETDRS) standard field 1 and ETDRS standard field 2, centered on the optic disc and fovea, respectively [34]. Retinal vascular caliber was measured with computer-assisted software according to standardized protocols [27], [35]–[37]. Figure S1 in File S1 highlights retinal arteriolar and venular calibers, summarized as CRAE and CRVE equivalent, respectively from retinal fundus photograph that were used in the study. Vessel measurements in each study were carried out by trained graders, masked to study subjects’ characteristics. Image of the right eye was used for measurements and when this was unavailable or un-gradable (due to disease or when at least 6 measurements of arterioles or venules could not be measured), the left eye image was examined. All arterioles and venules crossing through a specified zone 0.5–1 disc diameter away from the optic disc margin were measured and summarized as the CRAE or CRVE, respectively.

#### Exposure variables

Study participants’ demographic characteristics, lifestyle factors and medical history were obtained using a standardized questionnaire in the 3 Singaporean datasets. Age was defined as the age at the time of clinical examination. Cigarette smoking data was categorized into 2 categories as current smokers and former or non-smokers. Height in meters (m) and weight in kilograms (kg) were measured in all dataset and used to derive body-mass index (BMI). An average of at least two systolic blood pressures (SBP) and diastolic blood pressures (DBP) measurements was taken and hypertension was defined as a history of hypertension or SBP>140 mmHg or DBP>90 mmHg.

In SP2, fasting glucose and serum lipid concentrations were analyzed using kits from Boehringer Mannheim Systems (Mannheim, Germany) and read on a BM/Hitachi 747 analyzer (Roche Diagnostics, Corp. Indianapolis, USA). Glycosylated haemoglobin (HbA1c) levels and serum lipids were measured in non-fasting blood from SiMES and SINDI participants, using enzymatic methods implemented in the Advia 2400 Chemistry System (Siemens Medical Solutions Diagnostics, Deerfield, IL, USA). In the SINDI dataset, non-fasting HbA1c and serum lipids were measured with turbidimetric inhibition immonoassays using the Roche-Cobas® CE analyser (Roche Diagnostics, Germany), respectively. Diabetes was defined as participants with a history of diabetes mellitus in all datasets or fasting glucose levels ≥7.0 mmol/L in SP2 or HbA1c levels ≥6.5% in non-fasting blood samples in SiMES and SINDI.

### Statistical Analysis

#### GWAS association analysis with serum C-reactive protein

SNP-based trend tests for serum CRP associations were carried out in a chip-wise manner in the 3 Singaporean datasets. Associations between SNPs and CRP level were analysed in an additive model and adjusted for age, sex and population stratification (the first 2 principal components in SiMES and the first 3 principal components in SINDI only). These analyses were performed using the genome association toolset, SNPTEST (version 1.1.5) [32]. For analyses of imputed SNPs, SNP information scores were required to be at least 0.5. Genomic control (GC) correction was applied to association results from each chip, to further control for possible residual inflations.

Global genome-wide data from individual study results were subsequently combined (N = 6,692) using the inverse variance-weighted meta-analysis, assuming only a fixed effects model as the first aim was to identify novel loci. Cochran’s Q with a Q_p-value_ cut-off <0.1 was used to determine SNPs with between-study heterogeneity [37]. All meta-analysis procedures were performed using the *meta* package in STATA (version 8.2).

#### In silico replication analysis of previously reported candidate SNPs

All recently discovered index serum CRP-associated variants (35 SNPs from 22 loci) from GWAS studies [7]–[10] were selected for *in silico* replication among the 3 Singaporean datasets. 4 index SNPs [rs769449, rs3091244, rs1800961 and rs10521222 at the *APOE*, *CRP*, hepatocyte nuclear factor 4 (*HNF4A*) and sal-like 1 (*SALL1*) gene loci, respectively] were not genotyped or failed QC procedures or were not captured after imputation procedures in all 3 Singaporean datasets (Table S3 in File S1). At gene loci where multiple index SNPs were identified [*APOE*, *CRP*, glucokinase regulator (*GCKR*), *HNF1A*, interleukin 6 receptor (*IL6R*) and *LEPR*] pair-wise linkage disequilibrium (LD) was calculated using HapMap CHB and GIH panels. When LD between SNPs were high (r^2^>0.8 in both HapMap CHB and GIH panels), 1 tagging SNP was selected for *in silico* replication, prioritised if the SNP was actually genotyped in the Singapore datasets as compared to imputed SNPs (Table S3 in File S1).

To determine significance, consistency of effect direction with previously reported GWAS data was ensured in the Singaporean datasets and as these loci have been well-validated, a p-value threshold <0.05 was used. A Binomial test was used to assess over-representation of significant associations compared to the expected at α = 0.05. For all replicating index SNPs, we repeated the meta-analysis procedure using the more conservative random effects model to more accurately estimate effect sizes from the different ethnic groups (East-Asian Chinese and Malays and South-Asian Indian datasets) that had been combined. A Cochran’s Q_p-value_ cut-off <0.1 was used to determine SNPs with between-study heterogeneity [37]. Further secondary analyses adjusting for cardiovascular risk factors known to be associated with serum CRP (BMI, LDL-C and HDL-C levels and diabetes, hypertension and smoking status) [38]–[39] were also conducted to identify any modifications in association levels at these specific loci.

At loci that showed effects in opposite directions in our study (compared to initial European GWAS [7]), quantification of inter-population linkage disequilibrium (LD) differences was performed with varLD [40], using European (CEU) and East-Asian Chinese (CHB) data from HapMap [30] and Chinese, Malay and Asian-Indian reference panels from SGVP [31]. We further assessed for inter-population directional differences in LD by R correlation of regional SNPs (1 Mb region) in HapMap European (CEU), Han Chinese (CHB) and Indian panels (GIH).

#### Association analysis with retinal vessel traits

Association between serum CRP and retinal vessel traits (CRVE and CRAE) were first assessed in the Singaporean datasets. Next, one *CRP* variant that was genotyped in all Singaporean datasets and had the strongest association with serum CRP levels in our study was selected for assessment with retinal vessel traits (CRVE and CRAE). Regression analyses of CRVE and CRAE in SP2, SiMES and SINDI were further adjusted for age, sex and population stratification (the first two principal components in SiMES and the first three principal components in SINDI only), using STATA (version 8.2). Further validation of any significant association was sought from meta-analysis data of European populations that had recently completed a GWAS for retinal vessel traits [27].

We subsequently calculated predicted magnitude of effect, between the *CRP* variant and CRVE in the 3 Singaporean datasets. Predicted increase of CRVE per *CRP* risk allele was obtained by multiplying the observed beta of serum CRP (per *CRP* risk allele) with the observed beta of CRVE per SD (standard deviation) increase in serum CRP. Predicted and observed effect estimates were subsequently compared for the increase per risk allele. Power to detect the mean predicted increase in CRVE (from SP2, SiMES and SINDI datasets) among datasets was calculated using QUANTO [41].

## Results

### *CRP*, *APOE*, *HNF1A* and *LEPR* Variants were Associated with Serum C-reactive Protein in Singaporean Asian Datasets at Genome-wide Levels of Significance

Individual and meta-analysis GWAS results of SiMES, SP2 and SINDI Asian cohorts revealed serum CRP-associated SNPs in or near *CRP*, *APOE*, *HNF1A and LEPR* with genome-wide levels of significance (meta-analysis p-value between 4.74×10^−8^ and 1.90×10^−21^, Table S4 in File S1). SNPs at these four loci included the same index SNPs reported in recent GWAS studies [7]–[10], or were in at least moderate LD [r^2^>0.8 in Hapmap East-Asian (CHB and JPT) and Indian (GIH) panels] with index SNPs (rs11265260 and rs2794520 from *CRP*, rs2075650 and rs4420638 from *APOE*, rs7310409, rs1183910 and rs1169310 from *HNF1A* and rs1892534 from *LEPR*), corroborating the initial findings at these loci.

### Variants at 4 Additional Gene Loci were Associated with Serum C-reactive Protein and were Directionally Consistent in the *in silico* Assessment of Singaporean Asian Datasets

All recently identified serum CRP-associated variants [7]–[10] that were combined in a fixed effect meta-analysis are indicated in Table 2 (see Table S5 in File S1 for full table of all index SNPs in individual datasets and after meta-analysis). In addition to the *CRP*, *APOE*, *HNF1A* and *LEPR* loci, we detected significant associations with serum CRP level for index SNPs at 6 additional loci [*IL6R*, G protein-coupled receptor, family C, group 6, member A (*GPRC6A*), *GCKR*, interleukin 6 (*IL6*), interleukin 1 family, member 10 (*IL1F10*) and protein tyrosine phosphatase, non-receptor type 2 (*PTPN2*)] at more nominal levels of significance (meta-analyses p-values between 5.09×10^−5^ and 0.036) (Table 2). Forest plots of all significant loci detected among the Singaporean datasets are provided in Figure S2 in File S1.

**Table 2:** Association results of index CRP loci (7–10) in Singaporean datasets.

					SP2 (N = 2,179)			SiMES (N = 2,275)			SINDI (N = 2,238)			Fixed-effect meta-analysis (N = 6,692)		
rsid	Gene	Chr	Position	Test allele	TAF*	Beta	p-value	TAF	Beta	p-value	TAF	Beta	p-value	Beta	p-value	Q_p-value_
rs2075650	*APOE*	19	50087459	G	0.089	0.107	**1.98×10^−5^**	0.122	0.127	**1.02×10^−10^**	0.125	0.110	**7.07×10^−9^**	0.116	**1.90×10^−21^**	0.408
rs11265260	*CRP*	1	157966663	G	0.167	−0.122	**1.18×10^−10^**	0.113	−0.106	**4.13×10^−7^**	0.106	−0.071	**6.66×10^−4^**	−0.101	**1.13×10^−17^**	0.208
rs2794520	*CRP*	1	157945440	G	0.435	0.072	**3.76×10^−7^**	0.491	0.065	**4.50×10^−7^**	0.653	0.051	**1.64×10^−4^**	0.063	**8.88×10^−16^**	0.677
rs4420638	*APOE*	19	50114787	A	0.103	0.081	**5.42×10^−4^**	0.159	0.126	**1.54×10^−9^**	0.101	0.108	**4.14×10^−5^**	0.107	**1.55×10^−15^**	0.108
rs1183910	*HNF1A*	12	119905190	G	0.614	0.060	**5.56×10^−6^**	0.709	0.057	**8.09×10^−5^**	0.584	0.046	**3.96×10^−4^**	0.054	**3.55×10^−12^**	0.901
rs7310409	*HNF1A*	12	119909244	G	0.622	0.060	**3.01×10^−5^**	0.675	0.062	**7.54×10^−6^**	0.459	0.038	**0.003**	0.053	**2.85×10^−11^**	0.567
rs1169310	*HNF1A*	12	119923816	G	0.508	0.050	**7.91×10^−5^**	0.601	0.045	**7.65×10^−4^**	0.472	0.038	**0.003**	0.044	**2.61×10^−9^**	0.897
rs1892534	*LEPR*	1	65878532	T	0.877	−0.045	**0.036**	0.779	−0.067	**2.60×10^−5^**	0.496	−0.036	**0.005**	−0.047	**1.23×10^−7^**	0.480
rs4537545	*IL6R*	1	152685503	T	0.370	−0.053	**3.66×10^−4^**	0.224	−0.024	0.117	0.295	0.026	0.059	0.034	**5.09×10^−5^**	0.312
rs6901250^†^	*GPRC6A*	6	117220718	G	0.448	0.034	**0.009**	0.482	0.010	0.430	0.760	0.037	**0.015**	0.026	**0.008**	0.429
rs1260326	*GCKR*	2	27584444	T	0.478	0.021	0.144	0.394	0.020	0.132	0.201	0.026	0.101	0.022	**0.008**	0.919
rs2097677	*IL6*	7	22699364	G	0.863	−0.034	0.071	0.895	−0.045	**0.033**	0.778	−0.013	0.388	−0.027	**0.009**	0.656
rs6734238	*IL1F10*	2	113557501	A	0.083	−0.039	0.080	0.110	−0.038	0.074	0.362	−0.017	0.194	−0.026	**0.009**	0.510
rs2847281^†^	*PTPN2*	13	12811593	G	0.124	0.027	0.168	0.121	0.034	0.091	0.219	0.011	0.458	0.022	**0.036**	0.864
rs2836878	*PSMG1*	21	39387404	A	0.835	−0.021	0.267	0.861	−0.012	0.538	0.775	−0.024	0.108	−0.020	0.052	0.980
rs12239046	*NLRP3*	1	245668218	T	0.381	0.009	0.523	0.357	0.006	0.629	0.420	0.029	0.025	0.015	0.052	0.387
rs340029	*RORA*	15	58682257	T	0.903	−0.014	0.559	0.866	−0.016	0.398	0.608	−0.021	0.098	−0.019	0.054	0.993
rs10745954	*ASCL1*	12	102007224	G	0.794	0.023	0.140	0.823	−0.015	0.394	0.593	0.024	0.066	0.014	0.105	0.119
rs10778213		12	102019281	T	0.147	−0.014	0.470	0.184	0.015	0.384	0.451	−0.028	**0.026**	−0.013	0.147	**0.085**
rs4903031	*RGS6*	14	72088989	G	0.156	−0.043	**0.028**	0.249	0.014	0.343	0.327	−0.014	0.286	−0.010	0.258	**0.046**
rs4705952	*IRF1*	5	131867518	G	0.554	−0.013	0.366	0.462	0.005	0.728	0.396	−0.019	0.197	−0.009	0.279	0.405
rs13233571	*BCL7B*	7	72609167	T	0.092	0.008	0.709	0.117	−0.004	0.833	0.063	−0.035	0.187	−0.008	0.562	0.807

22 SNPs were genotyped or imputed and passed QC procedures in all 3 datasets and were combined in a meta-analysis (see Table S5 in File S1 for full results). Significant results (p-value <0.05) in bold.

TAF: Test allele frequency. ^†^ Variants which were significant but not directionally consistent with previous European study (7).

* Mean allele frequency from 3 SNP-chips used for the SP2 study. Q_pvalue_ <0.1 indicates between study heterogeneity.

At 2 variants, rs6901250 at *GPRC6A* and rs2847281 at *PTPN2* (Table 2), direction of effects were observed to be opposite in our Singaporean Asian datasets (as compared to the initial European GWAS [7]). varLD scores of pair-wise comparisons between different ethnic groups at these two gene regions did not indicate LD differences that were significantly greater than the entire genome (all regional scores<than 0.5 SD and thus, below the 67^th^ percentile of LD variations across the entire genome, Figure S3 in File S1). We also did not detect any contiguous regions with at least moderate LD (r correlation >0.6) with these 2 index SNPs that had opposite directions of effect when comparing European (CEU) and Chinese (CHB) or Indian (GIH) reference panels from HapMap, at the *GPRC6A* and *PTPN2* regions (data not shown).

Thus in total, we were able to replicate associations at 12 SNPs from 8 (*CRP*, *APOE*, *HNF1A*, *LEPR*, *IL6R*, *GCKR*, *IL6* and *IL1F10*) of the 22 known previously-reported CRP-associated gene loci with consistent beta directions, among the East-Asian and South-Asian study subjects participants from Singapore (Binomial-test p-value <0.001 for over-representation of significant results, α = 0.05). Assuming independence, these 12 SNPs explain approximately 4.05% of serum CRP variance in the Singaporean populations studied (Table 3). QQ-plot of meta-analysis p-value associations after excluding SNPs around (100 kb upstream and downstream) the 22 previously identified CRP-associated gene loci, did not show an excess of small p-values (Figure S4 in File S1).

**Table 3:** Additional association analyses for 12 index CRP loci (7–10) that were significant (fixed-effect meta-analysis p-value <0.05) in the Singaporean datasets and showed consistent direction of effect estimates.

					Fixed-effect meta-analysis*(N = 6,692)		Random-effect meta-analysis (N = 6,692)			
rsid	Gene	Chr	Position	Test allele	Beta*	p-value*	Beta	p-value	Q_p-value_	Variance explained (%)
rs2075650	*APOE*	19	50087459	G	0.124	**3.69×10^−24^**	0.116	**1.90×10^−21^**	0.408	0.70
rs11265260	*CRP*	1	157966663	G	−0.107	**3.54×10^−20^**	−0.100	**5.53×10^−11^**	0.208	0.68
rs2794520	*CRP*	1	157945440	G	0.069	**3.05×10^−19^**	0.063	**8.88×10^−16^**	0.677	0.45
rs4420638	*APOE*	19	50114787	A	0.123	**1.75×10^−22^**	0.107	**1.55×10^−15^**	0.108	0.62
rs1183910	*HNF1A*	12	119905190	G	0.061	**1.13×10^−16^**	0.054	**3.55×10^−12^**	0.901	0.33
rs7310409	*HNF1A*	12	119909244	G	0.060	**2.22×10^−16^**	0.053	**5.91×10^−11^**	0.567	0.30
rs1169310	*HNF1A*	12	119923816	G	0.048	**1.22×10^−12^**	0.044	**2.61×10^−9^**	0.897	0.26
rs1892534	*LEPR*	1	65878532	T	−0.045	**4.27×10^−8^**	−0.048	**1.15×10^−6^**	0.480	0.23
rs4537545	*IL6R*	1	152685503	T	−0.035	**8.09×10^−6^**	−0.034	**5.09×10^−5^**	0.312	0.20
rs1260326	*GCKR*	2	27584444	T	0.022	**0.008**	0.022	**0.008**	0.919	0.10
rs2097677	*IL6*	7	22699364	G	−0.032	**7.51×10^−4^**	−0.027	**0.009**	0.656	0.10
rs6734238	*IL1F10*	2	113557501	A	−0.023	**0.013**	0.026	**0.009**	0.510	0.08

Significant results (p-value <0.05) in bold.

* Additional covariates include BMI, BP, LDL-C, HDL-C and diabetes and smoking status. Q_pvalue_ <0.1 indicates between study heterogeneity. Variance explained: mean variance from all 3 Singaporean datasets.

Further assessment of these 12 loci through meta-analysis performed assuming the random effects model (at these specific SNPs) indicated reduced association levels at several SNPs (rs11265260, rs1892534 and rs7310409) but the same set of 12 SNPs remained significant (Table 3). Moreover, adjustment for other cardiovascular risk factors did not abolish these associations (p-value between 3.69×10^−24^ and 0.013, Table 3).

2 additional index variants, rs10778213 and rs4903031, showed heterogeneity (Q_pvalue_<0.1) in the meta-analysis of all 3 Sinagporean datasets (Table 2). For each of these variants, the direction of effect in the SiMES Malay subjects was opposite to that of the Chinese (SP2) and Asian-Indian (SINDI) ethnic groups.

### C-reactive Protein Increasing Variant (rs2794520) is not Associated with Retinal Microvascular Caliber

We selected one index *CRP* SNP, rs2794520, that had been directly genotyped in all Singaporean datasets and had the strongest association with serum CRP levels in our meta-analysis (rs2794520, p-value = 8.88×10^−16^ in both fixed and random-effect models, Tables 2 and 3), for assessment with CRVE and CRAE traits.

**Figure 1:**
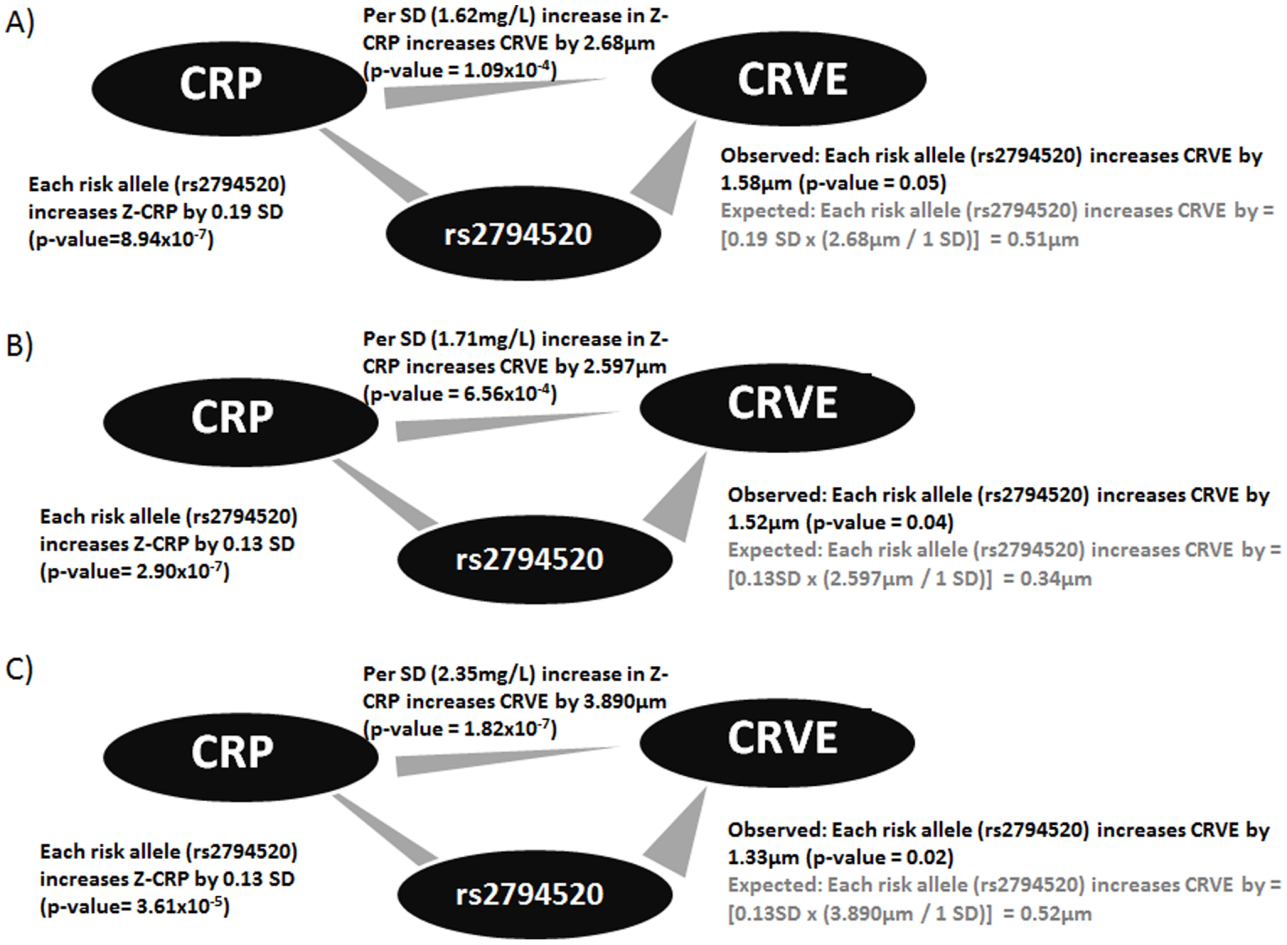
Comparison of observed and expected association results for rs2794520 and CRVE in A) SP2, B) SiMES and C) SINDI datasets. Observed regression values presented in black and estimated values presented in gray. Regression models were adjusted for age and sex in SP2 and age, sex and population stratification (first 2 principal components in SiMES and first 3 principal components in SINDI only).

In all the 3 Singaporean Asian datasets, serum CRP was associated with the CRVE phenotype (p-value ≤6.56×10^−4^, Figure 1) and the CRP increasing allele (G allele) of rs2794520 was marginally significant for increased CRVE (p-value between 0.048 and 0.022) but, this association was not replicated in the larger CHARGE consortium European samples and overall meta-analysis revealed no statistical significance (Table 4). No significant associations were detected for CRAE in any of the datasets (Table 4).

**Table 4:** Linear regression of rs2794520 with CRVE and CRAE traits in datasets used in the study.

Study	N	Estimated power (%)	MAF	Beta	SE	p-value	Q_p-value_
***CRVE association***							
SP2	1,411	16.0	0.435	1.578	0.778	0.048	
SiMES	2,230	20.4	0.491	1.520	0.716	0.038	
SINDI	2,390	21.3	0.653	1.326	0.610	0.022	
CHARGE	18,101	75.4	0.675	−0.006	0.231	0.978	
Fixed effect meta-analysis	24,132	89.2	NA	0.360	0.200	0.071	0.018
Random effect meta-analysis	24,132	89.2	NA	0.955	0.504	0.058	0.018
***CRVE association***							
SP2	1,413	NA	0.435	0.495	0.514	0.335	
SiMES	2,181	NA	0.491	0.365	0.487	0.454	
SINDI	2,390	NA	0.653	−0.326	0.447	0.466	
CHARGE	18,722	NA	0.675	−0.086	0.152	0.574	
Fixed effect meta-analysis	24,706	NA	NA	−0.034	0.132	0.796	0.517
Random effect meta-analysis	24,706	NA	NA	−0.034	0.132	0.796	0.517

The test allele for rs2794520 was the G allele.

Estimated power: based on average predicted effect estimate (0.45 µm CRVE) from SiMES, SP2 and SINDI datasets (see Figure 1) and a minor allele frequency of 0.40, at α = 0.05.

Power to detect changes of 0.45 µm in CRVE (mean predicted effect estimates in SP2, SiMES and SINDI datasets, Figure 1), assuming a minor allele frequency (MAF) of 40% (mean MAF of rs2794520 in SP2, SiMES and SINDI) (α = 0.05) was estimated to be low in individual Singaporean datasets and only adequate after meta-analysis of all Singaporean and European datasets (Table 4). Observed effect sizes for CRVE per increase in risk allele of rs2794520 were also 3–4 folds larger than expected among the Singaporean datasets (Figure 1).

## Discussion

Leveraging on GWAS data from 6,692 pan-Asian subjects, comprising of Chinese and Malay East-Asian and South-Asian Indian populations living in Singapore, we have shown that at least 8 genetic variants, initially identified in primarily European population studies, show similar associations with serum CRP. Furthermore, at these loci, genetic associations with serum CRP levels were observed to be independent of other known cardiovascular risk factors, while the replications of *LEPR*, *IL6R* and *IL1F10* among East-Asians and the replications of *GCKR*, *IL6* and *ILF10* among South-Asians were novel.

Marginal associations at 2 loci (rs6901250 at the *GPRC6A* locus and rs2847281 at the *PTPN2* locus) where we detected effect directions that were opposite to those identified in populations of European ancestry were likely to be false positive findings and our examination of inter-ethnic population LD differences did not reveal significant results. Further large-scale studies in East-Asian populations would be necessary to firmly confirm or dismiss associations at these loci.

A unique aspect of our study, in the assessment of causality with serum CRP, was the use of retinal vascular caliber (CRAE and CRVE), which are markers for systemic microvasculature and have been associated with subclinical and clinical cardiovascular disease [18]–[23]. Previous large-scale Mendelian randomisation studies did not detect an association between *CRP* variants and CHD or MI [7]–[8], [12]. Nevertheless, it is possible that the heterogeneous nature of such end-stage disease outcome measures may have masked true associations [42]. Furthermore, a recent association of *CRP* variants with CKD [16] highlighted the need to assess the role of CRP in microvascular disease, which has not been tested in previous studies.

In our study, although serum CRP was associated with CRVE levels, we do not find evidence to support a causal link between serum CRP and changes in retinal vascular caliber. It is noteworthy however, that marginally significant associations were detected between the *CRP* variant and CRVE in only the Singaporean Asian datasets (SP2 Chinese, SiMES Malays and SINDI Asian-Indians) but not among the European samples assessed. Although this may highlight an ethnic specific role, we were inadequately powered to detect such associations in the smaller Singaporean datasets and moreover, the association levels in the Singaporean datasets were nominal and observed CRVE effect sizes exceeded predicted estimates in all 3 Singaporean datasets, indicating that these associations may be stochastic. Furthermore, to prevent possible heterogeneity of assessing multiple SNPs, we limited this analysis to one index SNP (rs2794520 from the *CRP* gene locus), used as a proxy for serum CRP. However, although highly significant, this SNP only explained approximately 0.45% of the CRP phenotypic variance among the Singaporean datasets. Repeating the analysis using all 12 CRP-associated SNPs that replicated in the Singaporean meta-analysis (Table 2), with a gene-score method, did not improve the association between genetic variants and CRVE (data not shown).

A weakness of our study was the modest sample size for replication of serum CRP associations in East-Asian and South-Asian populations and it is likely that we lacked sufficient power to detect all true-positives, especially those with weaker effect sizes that were first detected only after large-scale meta-analyses (in European GWAS studies). Using an effect size of 1.35 mg/L increase in serum CRP (approximate effect size the *GPCR6A* and *PTPN2* variants in a recent large-scale European GWAS [7]), we estimated that a sample size of approximately 60,000 participants would be necessary to be at least 80% powered (α = 0.05) to detect variants of similar magnitude in our Singaporean Chinese, Malay and Asian-Indian datasets. Furthermore, although we increased power to detect associations by combining datasets in our meta-analyses, the diverse nature of the populations assessed may have contributed to between-study heterogeneity. However, it was reassuring that the same set of serum CRP-associated index SNPs remained statistically significant in the meta-analyses of datasets under the random effects model.

In conclusion, our meta-analysis of East-Asian and South-Asian subjects, who constitute the major portion of populations in Asia, wherein the burden of CVD is substantially increasing [43]–[44], replicates and corroborates at least 8 serum CRP-associated gene loci. However, we do not find evidence to support a causal link between serum CRP and markers for microvascular caliber.

## Supporting Information

File S1 **Combined supporting information file containing supporting tables and figure.** File includes: Table S1, Sample QC threshold of GWAS datasets used in study; Table S2, SNP QC thresholds utilised for GWAS datasets used in study; Table S3, Details of 35 index CRP SNPs identified from previous GWAS; Table S4, Genome-wide hits for serum CRP association after meta-analysis of SINDI, SP2 and SiMES; Table S5, Full association results of known CRP index variants in SP2, SiMES and SINDI datasets and after meta-analysis; Figure S1, Central retinal arteriolar equivalent and the central retinal venular equivalent from retinal fundus photograph using the Interactive Vessel Analysis; Figure S2, Forest plots comparing effect estimates from individual datasets and after fixed effect meta-analysis of all 14 SNPs that were observed to be significant for serum CRP association in the Singapore datasets; Figure S3, Inter-population pair-wise varLD comparison of Han Chinese population (CHB) and European population (CEU) from HapMap and Singaporean Chinese (CHS), Singaporean Malays (MAS) and Singaporean Indian populations from SGVP at the *GPRC6A* and *PTPN2* loci; Figure S4, QQ-plot for CRP association p-values of fixed-effect meta-analysis of all 3 Singaporean datasets (SP2, SiMES and SINDI) (N = 6,692) after removal of SNPs at 22 known CRP gene loci.
(DOC)
