# Supplementary material for: Are C-Reactive Protein Associated Genetic Variants Associated with Serum Levels and Retinal Markers of Microvascular Pathology in Asian Populations from Singapore?
Source: PLoS One. 2013 Jul 2;8(7):e67650. doi: 10.1371/journal.pone.0067650 (PMC3699653; doi:10.1371/journal.pone.0067650)

**Table S1:** Sample QC threshold of GWAS datasets used in study.

|  | **SINDI** | **SiMES** | **SP2** | | |
| --- | --- | --- | --- | --- | --- |
| SNP-chip | **610** | **610** | **1M** | **610** | **550** |
| Ethnicity | Asian-Indian | Malay | Chinese | | |
| *N* | 2953 | 3072 | 1016 | 1467 | 583 |
| Heterozygous and/or low call-rate* | 34 | 37 | 15 | 8 | 0 |
| Related samples and/or duplicates/contaminations | 326 | 279 | 28 | 287 | 13 |
| Discordant ethnic membership | 39 | 170 | 8 | 12 | 7 |
| Gender discrepancies | 16 | 44 | 12 | 14 | 1 |
| Remaining samples | 2538 | 2542 | 953 | 1146 | 562 |
| Samples with CRP measures* and genotype information | 2238 | 2275 | 830 | 1067 | 282 |

* Excluding 242 from SINDI, 190 samples from SiMES and 48 samples from SP2, respectively who had CRP values >10 mg/L.

**Table S2:** SNP QC thresholds utilised for GWAS datasets used in study.

| **Study** | **SINDI** | **SiMES** | **SP2** | | |
| --- | --- | --- | --- | --- | --- |
| Chip | Illumina 610 | Illumina 610 | Illumina 1M | Illumina 610 | Illumina 550 |
| Total Typed SNP | 620881 | 620881 | 1185068 | 620881 | 561466 |
| Non-autosomal (mitochondrial and sex chromosome) SNPs | 20431 | 20431 | 48765 | 20431 | 14008 |
| Typed SNPs with MAF < 0.05 | 59742 | 97814 | 185654 | 95793 | 74809 |
| Typed SNPs with HWE < 10^-4^ | 1041 | 558 | 118 | 50 | 30 |
| Typed SNPs with call-rate < 0.95 | 41331 | 42626 | 192586 | 58211 | 43277 |
| SNPs with allelic discrepancy between chips | - | - | 62 | | |
| Typed SNPs that pass QC | 498336 | 459452 | 757945 | 446334 | 429342 |
| Total Imputed SNPs | 1523736 | 1523530 | 1609626 | 1925335 | 1939848 |
| Imputed SNPs with MAF < 0.05 | 95779 | 172514 | 319199 | 374007 | 380729 |
| Imputed SNPs with HWE < 10^-4^ | 5868 | 4330 | 687 | 1519 | 231 |
| Imputed SNPs with call-rate < 0.95 | 502323 | 425808 | 225706 | 390866 | 398291 |
| Imputed SNPs with info score < 0.5 | 0 | 1 | 6 | 3 | 54 |
| Imputed SNPs that pass QC | 919766 | 920877 | 1064028 | 1158940 | 1160543 |
| Imputed and Typed SNPs pass QC | 1418102 | 1380329 | 1821973 | 16052747 | 1589885 |
| Merged SNPs | 1418102 | 1380329 | 1566423 | | |
| λ among QCed imputed and typed SNPs | 1.0106 | 1.0146 | 1.0121 | 1.0091 | 1.0119 |

**Table S3:** Details of 35 index CRP SNPs identified from previous GWAS. SNPs identified to be in poor LD with each other (r^2^ < 0.8) in HapMap Chinese (CHB) and Indian populations (GIH) indicated in bold.

| **rsid** | **Gene** | **Chr** | **Position** | **Comment** | **r^2^ in HapMap CHB panel** | **r^2^ in HapMap GIH panel** | **selected for *in silico* replication** |
| --- | --- | --- | --- | --- | --- | --- | --- |
| rs12037222 | *PABPC4* | 1 | 39837548 | typed |  |  | yes |
| rs6700896 | *LEPR* | 1 | 65862370 | imputed | 1.000 (rs6700896/rs1892534) | 0.977 (rs6700896/rs1892534) | no |
| rs1892534 |  | 1 | 65878532 | typed | 0.888 (rs6700896/rs4420065) | 0.977 (rs6700896/rs4420065) | yes |
| rs4420065 |  | 1 | 65934049 | typed | 0.893 (rs1892534/rs4420065) | 1.000 (rs1892534/rs4420065) | no |
| rs4537545 | *IL6R* | 1 | 1.53E+08 | typed | 0.952 (rs4537545/rs8192284) | 0.971 (rs4537545/rs8192284) | yes |
| rs4129267 |  | 1 | 1.53E+08 | typed | 1.000 (rs4129267/rs8192284) | 1.000 (rs4129267/rs8192284) | no |
| rs8192284 |  | 1 | 1.53E+08 | imputed | 0.952 (rs4537545/rs4129267) | 0.971 (rs4537545/rs4129267) | no |
| rs2794520 | *CRP* | 1 | 1.58E+08 | typed | **0.181 (rs2794520/rs11265260)** | **0.054 (rs2794520/rs11265260)** | yes |
| rs3091244 |  | 1 | 1.58E+08 | NA | **0.181 (rs7553007/rs11265260)** | **0.053 (rs7553007/rs11265260)** | no |
| rs3093059 |  | 1 | 1.58E+08 | imputed | **0.183 (rs3093059/rs7553007)** | **0.053 (rs3093059/rs7553007)** | no |
| rs7553007 |  | 1 | 1.58E+08 | imputed | 1.00 (rs2794520/rs7553007) | 0.976 (rs2794520/rs7553007) | no |
|  |  |  |  |  | 1.00 (rs3093059/rs11265260) | 1.00 (rs3093059/rs11265260) |  |
| rs11265260 |  | 1 | 1.58E+08 | imputed | **0.183 (rs2794520/rs3093059)** | **0.054 (rs2794520/rs3093059)** | yes |
| rs12239046 | *NLRP3* | 1 | 2.46E+08 | imputed |  |  | yes |
| rs1260326 | *GCKR* | 2 | 27584444 | typed | 0.926 (rs1260326/rs780094) | 0.964 (rs1260326/rs780094) | yes |
| rs780094 |  | 2 | 27594741 | typed |  |  | no |
| rs6734238 | *IL1F10* | 2 | 1.14E+08 | imputed |  |  | yes |
| rs4705952 | *IRF1* | 5 | 1.32E+08 | imputed |  |  | yes |
| rs6901250 | *GPRC6A* | 6 | 1.17E+08 | imputed |  |  | yes |
| rs2097677 | *IL6* | 7 | 22699364 | imputed |  |  | yes |
| rs13233571 | *BCL7B* | 7 | 72609167 | imputed |  |  | yes |
| rs9987289 | *PPP1R3B* | 8 | 9220768 | imputed |  |  | yes |
| rs10745954 | *ASCL1* | 12 | 1.02E+08 | imputed |  |  | yes |
| rs10778213 | *-* | 12 | 1.02E+08 | typed |  |  | yes |
| rs1183910 | *HNF1A* | 12 | 1.2E+08 | imputed | **0.742 (rs7310409/rs1169310)** | 0.830 (rs7310409/rs1169310) | yes |
| rs7310409 |  | 12 | 1.2E+08 | typed | **0.690 (rs7310409/rs1182910)** | **0.700 (rs7310409/rs1182910)** | yes |
| rs1169310 |  | 12 | 1.2E+08 | imputed | 0.929 (rs1169310/rs1182910) | **0.775 (rs1169310/rs1182910)** | yes |
| rs2847281 | *PTPN2* | 13 | 12811593 | imputed |  |  | yes |
| rs4903031 | *RGS6* | 14 | 72088989 | typed |  |  | yes |
| rs340029 | *RORA* | 15 | 58682257 | typed |  |  | yes |
| rs10521222 | *SALL1* | 16 | 49716211 | NA |  |  | no |
| rs2075650 | *APOE* | 19 | 50087459 | typed | **0.031 (rs2075650/rs4420638)** | **0.185 (rs2075650/rs4420638)** | yes |
| rs769449 |  | 19 | 50101843 | NA |  |  | no |
| rs4420638 |  | 19 | 50114787 | typed |  |  | yes |
| rs1800961 | *HNF4A* | 20 | 42475778 | MAF < 0.05 |  |  | no |
| rs2836878 | *PSMG1* | 21 | 39387404 | typed |  |  | yes |

NA: not genotyped or imputed in any dataset. MAF: minor allele frequency

**Table S4:** Genome-wide (p-value < 5x10^-8^) hits for serum CRP association after meta-analysis of SINDI, SP2 and SiMES. 85 SNPs from *APOE*, *CRP*, *HNF1A* and *LEPR* gene loci reached genome-wide levels in the meta-analysis.

|  |  |  |  |  |  | **SINDI**  **(N = 2,238)** | | | **SP2**  **(N = 2,179)** | | | **SiMES**  **(N = 2,275)** | | | **Fixed effect meta-analysis**  **(N = 6,692)** | | |
| --- | --- | --- | --- | --- | --- | --- | --- | --- | --- | --- | --- | --- | --- | --- | --- | --- | --- |
| **SNP** | **Chr** | **Position** | **Gene** | **Remark** | **TA** | **TAF** | **Beta** | **p-value** | **TAF*** | **Beta** | **p-value** | **TAF** | **Beta** | **p-value** | **Beta** | **p-value** | **Q_pvalue_** |
| rs2075650 | 19 | 50087459 | *APOE* | typed | G | 0.125 | 0.110 | 5.94E-09 | 0.089 | 0.107 | 1.98E-05 | 0.122 | 0.127 | 9.14E-11 | 0.116 | 1.90E-21 | 0.408 |
| rs16842559 | 1 | 157942795 | *CRP* | imp | T | 0.894 | 0.074 | 3.64E-04 | 0.823 | 0.124 | 1.30E-13 | 0.887 | 0.108 | 1.75E-07 | 0.106 | 4.10E-21 | 0.210 |
| rs16842568 | 1 | 157942844 | *CRP* | imp | G | 0.106 | -0.074 | 3.67E-04 | 0.176 | -0.124 | 1.30E-13 | 0.113 | -0.108 | 1.75E-07 | -0.106 | 4.15E-21 | 0.210 |
| rs16842502 | 1 | 157920487 | *CRP* | imp | C | 0.896 | 0.076 | 3.39E-04 | 0.821 | 0.122 | 3.46E-13 | 0.888 | 0.111 | 1.17E-07 | 0.106 | 5.56E-21 | 0.229 |
| rs16842599 | 1 | 157964099 | *CRP* | typed | T | 0.892 | 0.068 | 9.50E-04 | 0.824 | 0.129 | 5.20E-14 | 0.886 | 0.107 | 2.08E-07 | 0.105 | 1.15E-20 | 0.145 |
| rs3093075 | 1 | 157946537 | *CRP* | typed | T | 0.109 | -0.069 | 7.48E-04 | 0.179 | -0.125 | 1.54E-13 | 0.114 | -0.108 | 1.63E-07 | -0.104 | 1.45E-20 | 0.211 |
| rs12081252 | 1 | 157973137 | *CRP* | imp | T | 0.893 | 0.068 | 9.53E-04 | 0.824 | 0.126 | 7.62E-14 | 0.886 | 0.107 | 2.45E-07 | 0.104 | 1.47E-20 | 0.158 |
| rs12081480 | 1 | 157973614 | *CRP* | typed | T | 0.908 | 0.084 | 3.60E-04 | 0.823 | 0.129 | 7.31E-14 | 0.889 | 0.105 | 1.18E-06 | 0.111 | 1.98E-20 | 0.370 |
| rs1205 | 1 | 157948857 | *CRP* | typed | T | 0.332 | 0.053 | 1.04E-04 | 0.561 | 0.088 | 1.11E-11 | 0.517 | 0.067 | 2.34E-07 | 0.069 | 2.61E-20 | 0.179 |
| rs6857 | 19 | 50084094 | *APOE* | imp | T | 0.122 | 0.113 | 9.90E-09 | 0.088 | 0.102 | 9.10E-06 | 0.123 | 0.122 | 2.56E-09 | 0.113 | 3.73E-20 | 0.304 |
| rs12068753 | 1 | 157959161 | *CRP* | imp | T | 0.888 | 0.065 | 0.001 | 0.823 | 0.125 | 7.86E-14 | 0.885 | 0.105 | 3.91E-07 | 0.102 | 4.19E-20 | 0.127 |
| rs12081264 | 1 | 157973184 | *CRP* | typed | T | 0.890 | 0.065 | 0.001 | 0.825 | 0.129 | 4.93E-14 | 0.885 | 0.104 | 5.45E-07 | 0.103 | 4.94E-20 | 0.117 |
| rs2808629 | 1 | 157943420 | *CRP* | typed | G | 0.666 | -0.052 | 1.17E-04 | 0.438 | -0.087 | 1.86E-11 | 0.483 | -0.066 | 2.49E-07 | -0.069 | 4.97E-20 | 0.174 |
| rs7553007 | 1 | 157965173 | *CRP* | imp | G | 0.667 | -0.054 | 7.65E-05 | 0.440 | -0.084 | 5.43E-11 | 0.480 | -0.067 | 2.20E-07 | -0.069 | 5.09E-20 | 0.139 |
| rs2808628 | 1 | 157942635 | *CRP* | imp | G | 0.663 | -0.053 | 9.50E-05 | 0.440 | -0.083 | 7.01E-11 | 0.483 | -0.066 | 2.70E-07 | -0.068 | 1.07E-19 | 0.170 |
| rs876537 | 1 | 157941557 | *CRP* | typed | T | 0.360 | 0.048 | 3.66E-04 | 0.561 | 0.087 | 2.12E-11 | 0.519 | 0.067 | 1.88E-07 | 0.068 | 1.80E-19 | 0.145 |
| rs11265257 | 1 | 157935608 | *CRP* | imp | T | 0.363 | 0.048 | 4.06E-04 | 0.559 | 0.084 | 5.29E-11 | 0.519 | 0.068 | 1.30E-07 | 0.067 | 2.75E-19 | 0.127 |
| rs2808624 | 1 | 157932545 | *CRP* | imp | G | 0.363 | 0.048 | 3.95E-04 | 0.560 | 0.084 | 7.12E-11 | 0.519 | 0.069 | 1.10E-07 | 0.067 | 2.96E-19 | 0.145 |
| rs1341665 | 1 | 157958183 | *CRP* | imp | G | 0.652 | -0.052 | 1.22E-04 | 0.417 | -0.082 | 1.55E-10 | 0.476 | -0.066 | 3.42E-07 | -0.067 | 3.75E-19 | 0.228 |
| rs2027471 | 1 | 157956012 | *CRP* | imp | T | 0.649 | -0.052 | 1.23E-04 | 0.418 | -0.082 | 1.55E-10 | 0.474 | -0.066 | 3.54E-07 | -0.067 | 3.95E-19 | 0.232 |
| rs1470515 | 1 | 157920223 | *CRP* | imp | T | 0.364 | 0.047 | 4.13E-04 | 0.561 | 0.083 | 1.15E-10 | 0.518 | 0.069 | 9.91E-08 | 0.067 | 4.33E-19 | 0.159 |
| rs11265260 | 1 | 157966663 | *CRP* | typed | G | 0.106 | -0.071 | 6.25E-04 | 0.167 | -0.122 | 1.18E-10 | 0.114 | -0.106 | 3.85E-07 | -0.101 | 1.12E-17 | 0.208 |
| rs3093077 | 1 | 157946260 | *CRP* | typed | C | 0.114 | -0.064 | 0.001 | 0.173 | -0.115 | 8.34E-10 | 0.115 | -0.109 | 1.51E-07 | -0.097 | 5.14E-17 | 0.254 |
| rs12567054 | 1 | 157911592 | *CRP* | imp | T | 0.896 | 0.066 | 0.002 | 0.839 | 0.115 | 7.70E-11 | 0.901 | 0.100 | 1.02E-05 | 0.097 | 2.22E-16 | 0.213 |
| rs2794520 | 1 | 157945440 | *CRP* | typed | G | 0.334 | 0.051 | 1.52E-04 | 0.564 | 0.072 | 3.76E-07 | 0.518 | 0.065 | 4.20E-07 | 0.063 | 8.88E-16 | 0.677 |
| rs4420638 | 19 | 50114786 | *APOE* | typed | G | 0.101 | 0.108 | 3.79E-05 | 0.103 | 0.081 | 5.42E-04 | 0.159 | 0.126 | 1.39E-09 | 0.107 | 1.55E-15 | 0.108 |
| rs2592887 | 1 | 157919563 | *CRP* | typed | T | 0.362 | 0.047 | 4.20E-04 | 0.570 | 0.067 | 2.71E-06 | 0.506 | 0.066 | 3.11E-07 | 0.060 | 1.38E-14 | 0.551 |
| rs157582 | 19 | 50088059 | *APOE* | typed | T | 0.180 | 0.096 | 9.20E-08 | 0.188 | 0.065 | 1.09E-04 | 0.257 | 0.059 | 1.01E-04 | 0.071 | 7.57E-14 | 0.463 |
| rs4275453 | 1 | 157974671 | *CRP* | imp | T | 0.580 | 0.034 | 0.013 | 0.654 | 0.075 | 3.53E-08 | 0.648 | 0.062 | 1.15E-05 | 0.057 | 8.88E-13 | 0.155 |
| rs1935193 | 1 | 157930714 | *CRP* | imp | T | 0.400 | -0.032 | 0.016 | 0.345 | -0.074 | 9.55E-08 | 0.366 | -0.064 | 3.17E-06 | -0.056 | 1.07E-12 | 0.100 |
| rs1183910 | 12 | 119905190 | *HNF1A* | imp | G | 0.584 | 0.046 | 3.70E-04 | 0.614 | 0.060 | 5.56E-06 | 0.708 | 0.057 | 7.75E-05 | 0.054 | 3.55E-12 | 0.901 |
| rs12049404 | 1 | 157980468 | *CRP* | imp | T | 0.252 | 0.042 | 0.004 | 0.605 | 0.066 | 3.60E-07 | 0.518 | 0.051 | 7.32E-05 | 0.054 | 3.68E-12 | 0.204 |
| rs2244608 | 12 | 119901371 | *HNF1A* | imp | G | 0.423 | 0.047 | 4.47E-04 | 0.380 | 0.061 | 4.48E-06 | 0.299 | 0.055 | 1.75E-04 | 0.054 | 7.61E-12 | 0.896 |
| rs1811472 | 1 | 157908973 | *CRP* | typed | G | 0.637 | -0.040 | 0.002 | 0.229 | -0.091 | 1.55E-08 | 0.243 | -0.050 | 8.51E-04 | -0.057 | 1.21E-11 | 0.441 |
| rs2393791 | 12 | 119908339 | *HNF1A* | imp | T | 0.458 | -0.036 | 0.005 | 0.612 | -0.060 | 4.78E-06 | 0.675 | -0.061 | 1.13E-05 | -0.052 | 1.23E-11 | 0.616 |
| rs2393775 | 12 | 119908957 | *HNF1A* | imp | G | 0.543 | 0.036 | 0.005 | 0.391 | 0.059 | 5.87E-06 | 0.328 | 0.060 | 1.40E-05 | 0.051 | 1.82E-11 | 0.645 |
| rs2649999 | 12 | 119864927 | *HNF1A* | imp | T | 0.431 | 0.050 | 5.75E-04 | 0.437 | 0.062 | 1.94E-05 | 0.351 | 0.058 | 8.56E-05 | 0.056 | 1.83E-11 | 0.957 |
| rs11065385 | 12 | 119907769 | *HNF1A* | imp | G | 0.593 | 0.043 | 9.71E-04 | 0.621 | 0.057 | 1.82E-05 | 0.717 | 0.059 | 5.99E-05 | 0.052 | 2.49E-11 | 0.824 |
| rs10119 | 19 | 50098513 | *APOE* | imp | G | 0.808 | -0.036 | 0.065 | 0.911 | -0.123 | 4.46E-07 | 0.826 | -0.094 | 8.19E-07 | -0.080 | 2.51E-11 | 0.106 |
| rs7310409 | 12 | 119909244 | *HNF1A* | typed | G | 0.459 | 0.038 | 0.003 | 0.622 | 0.060 | 3.01E-05 | 0.675 | 0.062 | 7.14E-06 | 0.053 | 2.85E-11 | 0.567 |
| rs2650000 | 12 | 119873345 | *HNF1A* | typed | C | 0.574 | 0.051 | 6.67E-05 | 0.555 | 0.049 | 4.99E-04 | 0.652 | 0.054 | 5.85E-05 | 0.052 | 3.12E-11 | 0.964 |
| rs10437339 | 1 | 157977009 | *CRP* | imp | G | 0.535 | 0.037 | 0.004 | 0.674 | 0.070 | 3.50E-07 | 0.641 | 0.048 | 3.57E-04 | 0.051 | 3.43E-11 | 0.270 |
| rs2251468 | 12 | 119889509 | *HNF1A* | imp | C | 0.426 | 0.051 | 1.05E-04 | 0.396 | 0.049 | 1.60E-04 | 0.318 | 0.053 | 1.41E-04 | 0.051 | 3.76E-11 | 0.967 |
| rs2708104 | 12 | 119968332 | *HNF1A* | typed | T | 0.238 | -0.063 | 3.22E-05 | 0.405 | -0.033 | 0.021 | 0.568 | -0.061 | 2.71E-06 | -0.053 | 9.32E-11 | 0.438 |
| rs1182933 | 12 | 119939005 | *HNF1A* | typed | T | 0.431 | 0.046 | 5.28E-04 | 0.491 | 0.051 | 1.11E-04 | 0.359 | 0.050 | 2.97E-04 | 0.049 | 2.61E-10 | 0.980 |
| rs1572970 | 1 | 157940209 | *CRP* | typed | G | 0.404 | -0.032 | 0.014 | 0.362 | -0.058 | 7.36E-05 | 0.374 | -0.061 | 5.52E-06 | -0.050 | 3.17E-10 | 0.386 |
| rs3213545 | 12 | 119955720 | *HNF1A* | typed | G | 0.570 | -0.050 | 1.39E-04 | 0.534 | -0.046 | 4.86E-04 | 0.659 | -0.051 | 3.26E-04 | -0.049 | 3.22E-10 | 0.992 |
| rs10774580 | 12 | 119960806 | *HNF1A* | typed | G | 0.724 | 0.054 | 1.37E-04 | 0.594 | 0.032 | 0.025 | 0.410 | 0.061 | 4.24E-06 | 0.050 | 5.53E-10 | 0.517 |
| rs7953249 | 12 | 119888107 | *HNF1A* | typed | G | 0.572 | 0.044 | 7.32E-04 | 0.444 | 0.050 | 3.73E-04 | 0.364 | 0.051 | 1.55E-04 | 0.048 | 6.39E-10 | 0.930 |
| rs7979473 | 12 | 119904643 | *HNF1A* | typed | G | 0.442 | -0.036 | 0.007 | 0.611 | -0.060 | 6.55E-06 | 0.628 | -0.047 | 5.25E-04 | -0.048 | 6.91E-10 | 0.679 |
| rs7979478 | 12 | 119904646 | *HNF1A* | typed | G | 0.442 | -0.036 | 0.007 | 0.611 | -0.060 | 6.77E-06 | 0.628 | -0.047 | 5.25E-04 | -0.048 | 7.07E-10 | 0.682 |
| rs4285692 | 1 | 157980773 | *CRP* | imp | T | 0.466 | -0.037 | 0.004 | 0.319 | -0.063 | 3.63E-06 | 0.369 | -0.043 | 1.27E-03 | -0.047 | 7.28E-10 | 0.350 |
| rs1169312 | 12 | 119925844 | *HNF1A* | typed | T | 0.538 | -0.043 | 9.56E-04 | 0.495 | -0.050 | 1.29E-04 | 0.404 | -0.045 | 8.04E-04 | -0.046 | 1.27E-09 | 0.964 |
| rs2259820 | 12 | 119919725 | *HNF1A* | typed | T | 0.431 | 0.041 | 0.001 | 0.488 | 0.051 | 8.56E-05 | 0.359 | 0.045 | 1.02E-03 | 0.046 | 1.32E-09 | 0.924 |
| rs1169313 | 12 | 119927053 | *HNF1A* | typed | T | 0.472 | 0.038 | 0.002 | 0.509 | 0.051 | 7.00E-05 | 0.601 | 0.045 | 6.73E-04 | 0.045 | 1.58E-09 | 0.897 |
| rs1169306 | 12 | 119922694 | *HNF1A* | imp | T | 0.529 | -0.038 | 0.003 | 0.492 | -0.050 | 7.92E-05 | 0.400 | -0.045 | 7.56E-04 | -0.044 | 2.40E-09 | 0.890 |
| rs2259816 | 12 | 119919970 | *HNF1A* | typed | T | 0.525 | -0.038 | 0.003 | 0.490 | -0.051 | 8.08E-05 | 0.399 | -0.044 | 9.60E-04 | -0.044 | 2.59E-09 | 0.888 |
| rs1169310 | 12 | 119923816 | *HNF1A* | imp | G | 0.472 | 0.038 | 0.003 | 0.508 | 0.050 | 7.91E-05 | 0.600 | 0.045 | 7.41E-04 | 0.044 | 2.61E-09 | 0.897 |
| rs2464195 | 12 | 119919858 | *HNF1A* | imp | G | 0.476 | 0.038 | 0.003 | 0.509 | 0.050 | 9.92E-05 | 0.602 | 0.044 | 9.76E-04 | 0.044 | 3.08E-09 | 0.901 |
| rs1169300 | 12 | 119915608 | *HNF1A* | typed | G | 0.570 | -0.041 | 0.002 | 0.518 | -0.048 | 7.58E-04 | 0.644 | -0.049 | 3.24E-04 | -0.046 | 4.56E-09 | 0.848 |
| rs11588887 | 1 | 157983786 | *CRP* | typed | G | 0.749 | -0.040 | 0.007 | 0.390 | -0.053 | 2.67E-04 | 0.481 | -0.049 | 1.85E-04 | -0.047 | 5.35E-09 | 0.925 |
| rs1446976 | 1 | 157895818 | *CRP* | imp | G | 0.267 | -0.032 | 0.026 | 0.178 | -0.093 | 1.19E-08 | 0.120 | -0.039 | 0.050 | -0.054 | 5.84E-09 | 0.106 |
| rs2464196 | 12 | 119919810 | *HNF1A* | typed | G | 0.569 | -0.041 | 0.001 | 0.517 | -0.048 | 7.32E-04 | 0.640 | -0.047 | 5.55E-04 | -0.045 | 6.13E-09 | 0.845 |
| rs1891187 | 1 | 157895677 | *CRP* | imp | T | 0.733 | 0.031 | 0.029 | 0.822 | 0.093 | 1.19E-08 | 0.881 | 0.038 | 0.053 | 0.054 | 7.32E-09 | 0.104 |
| rs10774579 | 12 | 119889593 | *HNF1A* | typed | T | 0.634 | -0.037 | 0.006 | 0.394 | -0.047 | 0.001 | 0.360 | -0.053 | 9.28E-05 | -0.045 | 8.50E-09 | 0.852 |
| rs10889569 | 1 | 65858782 | *LEPR* | imp | T | 0.490 | 0.038 | 0.003 | 0.874 | 0.061 | 0.002 | 0.778 | 0.062 | 8.99E-05 | 0.050 | 8.53E-09 | 0.530 |
| rs6588153 | 1 | 65864605 | *LEPR* | imp | T | 0.498 | -0.038 | 0.003 | 0.126 | -0.060 | 0.002 | 0.221 | -0.064 | 6.46E-05 | -0.051 | 8.74E-09 | 0.518 |
| rs6678033 | 1 | 65850212 | *LEPR* | typed | G | 0.505 | -0.041 | 0.002 | 0.126 | -0.058 | 0.003 | 0.223 | -0.062 | 1.09E-04 | -0.051 | 1.19E-08 | 0.599 |
| rs7531867 | 1 | 65880134 | *LEPR* | imp | G | 0.505 | -0.035 | 0.006 | 0.126 | -0.060 | 0.002 | 0.218 | -0.065 | 3.99E-05 | -0.049 | 1.19E-08 | 0.404 |
| rs2258844 | 12 | 119972755 | *HNF1A* | imp | G | 0.227 | 0.050 | 0.002 | 0.402 | 0.035 | 0.009 | 0.561 | 0.055 | 2.59E-05 | 0.046 | 1.19E-08 | 0.611 |
| rs735396 | 12 | 119923227 | *HNF1A* | typed | T | 0.470 | -0.039 | 0.002 | 0.514 | -0.048 | 7.19E-04 | 0.600 | -0.046 | 6.78E-04 | -0.044 | 1.21E-08 | 0.787 |
| rs1169286 | 12 | 119903439 | *HNF1A* | typed | T | 0.506 | -0.041 | 0.002 | 0.532 | -0.052 | 6.91E-05 | 0.565 | -0.036 | 9.64E-03 | -0.043 | 1.52E-08 | 0.765 |
| rs2794526 | 1 | 157892102 | *CRP* | imp | G | 0.726 | 0.033 | 0.024 | 0.825 | 0.095 | 1.36E-08 | 0.869 | 0.033 | 0.098 | 0.053 | 1.56E-08 | 0.103 |
| rs7516341 | 1 | 65860731 | *LEPR* | typed | T | 0.510 | -0.038 | 0.003 | 0.126 | -0.058 | 0.003 | 0.222 | -0.063 | 8.37E-05 | -0.050 | 1.57E-08 | 0.580 |
| rs4655557 | 1 | 65853375 | *LEPR* | typed | T | 0.508 | -0.039 | 0.002 | 0.126 | -0.058 | 0.003 | 0.223 | -0.062 | 1.06E-04 | -0.050 | 1.58E-08 | 0.607 |
| rs157580 | 19 | 50087106 | *APOE* | typed | G | 0.506 | -0.049 | 8.75E-05 | 0.558 | -0.044 | 0.002 | 0.447 | -0.036 | 5.92E-03 | -0.043 | 1.76E-08 | 0.758 |
| rs12042779 | 1 | 65906538 | *LEPR* | imp | T | 0.505 | -0.034 | 0.006 | 0.126 | -0.060 | 0.002 | 0.214 | -0.064 | 6.04E-05 | -0.049 | 1.78E-08 | 0.397 |
| rs12042807 | 1 | 65906640 | *LEPR* | imp | T | 0.505 | -0.034 | 0.006 | 0.126 | -0.060 | 0.002 | 0.214 | -0.064 | 6.04E-05 | -0.049 | 1.78E-08 | 0.397 |
| rs10789192 | 1 | 65898358 | *LEPR* | imp | G | 0.505 | -0.034 | 0.006 | 0.126 | -0.060 | 0.002 | 0.214 | -0.064 | 6.04E-05 | -0.049 | 1.78E-08 | 0.397 |
| rs1805096 | 1 | 65874845 | *LEPR* | typed | G | 0.505 | -0.035 | 0.006 | 0.126 | -0.057 | 0.004 | 0.219 | -0.065 | 3.92E-05 | -0.048 | 2.25E-08 | 0.456 |
| rs6700896 | 1 | 65862370 | *LEPR* | typed | T | 0.498 | 0.036 | 0.005 | 0.874 | 0.056 | 0.004 | 0.780 | 0.064 | 6.14E-05 | 0.049 | 2.71E-08 | 0.517 |
| rs11208711 | 1 | 65920434 | *LEPR* | imp | T | 0.494 | 0.034 | 0.007 | 0.874 | 0.060 | 0.002 | 0.784 | 0.061 | 1.20E-04 | 0.048 | 3.23E-08 | 0.439 |
| rs4655584 | 1 | 65928103 | *LEPR* | imp | T | 0.506 | -0.034 | 0.007 | 0.126 | -0.060 | 0.002 | 0.216 | -0.061 | 1.27E-04 | -0.048 | 3.33E-08 | 0.443 |
| rs4655585 | 1 | 65928215 | *LEPR* | imp | T | 0.506 | -0.034 | 0.007 | 0.126 | -0.060 | 0.002 | 0.217 | -0.061 | 1.37E-04 | -0.048 | 3.53E-08 | 0.448 |
| rs7305618 | 12 | 119887315 | *HNF1A* | typed | T | 0.297 | 0.023 | 0.094 | 0.555 | 0.051 | 3.22E-04 | 0.597 | 0.055 | 3.05E-05 | 0.044 | 4.70E-08 | 0.436 |
| rs11265265 | 1 | 157980180 | *CRP* | typed | T | 0.466 | -0.038 | 0.003 | 0.317 | -0.053 | 4.08E-04 | 0.368 | -0.040 | 2.87E-03 | -0.043 | 4.74E-08 | 0.907 |

Typed: SNP genotyped in at least 1 dataset; imputed: imputed SNP; TA: test allele, TAF: test allele frequency.

* Mean allele frequency from 3 SNP-chip data of SP2 cohort.

**Table S5:** Full association results of known CRP index variants (7-10) in SP2, SiMES and SINDI datasets and after meta-analysis (fixed effects). 22 SNPs were genotyped or imputed and passed QC procedures in all 3 datasets and were combined in a meta-analysis. Significant results (P-value < 0.05) in bold. 2 SNPs (rs10778213and rs4903031) showed significant between-study heterogeneity (Q_pvalue_ < 0.1).

|  |  |  |  |  |  | **SINDI**  **(N = 2,238)** | | | | **SP2**  **(N = 2,179)** | | | | **SiMES**  **(N = 2,275)** | | | | **Fixed effect meta-analysis**  **(N = 6,692)** | | |
| --- | --- | --- | --- | --- | --- | --- | --- | --- | --- | --- | --- | --- | --- | --- | --- | --- | --- | --- | --- | --- |
| **rsid** | **Gene** | **Chr** | **Position** | **Remark** | **TA** | **TAF** | **Beta** | **SE** | **p-value** | **TAF*** | **Beta** | **SE** | **p-value** | **TAF** | **Beta** | **SE** | **p-value** | **Beta** | **p-value** | **Q_pvalue_** |
| rs2075650 | *APOE* | 19 | 50087459 | typed | G | 0.125 | 0.110 | 0.019 | **7.07x10^-9^** | 0.089 | 0.107 | 0.025 | **1.98x10^-5^** | 0.122 | 0.127 | 0.020 | **1.02x10^-10^** | 0.116 | **1.90x10^-21^** | 0.408 |
| rs11265260 | *CRP* | 1 | 157966663 | imp | G | 0.106 | -0.071 | 0.021 | **6.66 x10^-4^** | 0.167 | -0.122 | 0.019 | **1.18x10^-10^** | 0.113 | -0.106 | 0.021 | **4.13x10^-7^** | -0.101 | **1.13x10^-17^** | 0.208 |
| rs2794520 | *CRP* | 1 | 157945440 | typed | G | 0.653 | 0.051 | 0.014 | **1.64 x10^-4^** | 0.435 | 0.072 | 0.014 | **3.76 x10^-7^** | 0.491 | 0.065 | 0.013 | **4.50x10^-7^** | 0.063 | **8.88x10^-16^** | 0.677 |
| rs4420638 | *APOE* | 19 | 50114787 | typed | A | 0.101 | 0.108 | 0.026 | **4.14 x10^-5^** | 0.103 | 0.081 | 0.023 | **5.42x10^-10^** | 0.159 | 0.126 | 0.021 | **1.54x10^-9^** | 0.107 | **1.55x10^-15^** | 0.108 |
| rs1183910 | *HNF1A* | 12 | 119905190 | imp | G | 0.584 | 0.046 | 0.013 | **3.96 x10^-4^** | 0.614 | 0.060 | 0.013 | **5.56 x10^-6^** | 0.709 | 0.057 | 0.014 | **8.09x10^-5^** | 0.054 | **3.55x10^-12^** | 0.901 |
| rs7310409 | *HNF1A* | 12 | 119909244 | typed | G | 0.459 | 0.038 | 0.013 | **0.003** | 0.622 | 0.060 | 0.014 | **3.01 x10^-5^** | 0.675 | 0.062 | 0.014 | **7.54x10^-6^** | 0.053 | **2.85x10^-11^** | 0.567 |
| rs1169310 | *HNF1A* | 12 | 119923816 | imp | G | 0.472 | 0.038 | 0.013 | **0.003** | 0.508 | 0.050 | 0.013 | **7.91 x10^-5^** | 0.601 | 0.045 | 0.013 | **7.65x10^-4^** | 0.044 | **2.61x10^-9^** | 0.897 |
| rs1892534 | *LEPR* | 1 | 65878532 | typed | T | 0.496 | -0.036 | 0.013 | **0.005** | 0.877 | -0.045 | 0.021 | **0.036** | 0.779 | -0.067 | 0.016 | **2.60x10^-5^** | -0.047 | **1.23x10^-7^** | 0.480 |
| rs4537545 | *IL6R* | 1 | 152685503 | typed | T | 0.295 | -0.026 | 0.014 | **0.059** | 0.370 | -0.053 | 0.015 | **3.66 x10^-4^** | 0.224 | -0.024 | 0.015 | 0.117 | -0.034 | **5.09x10^-5^** | 0.312 |
| rs6901250 | *GPRC6A* | 6 | 117220718 | imp | G | 0.760 | 0.037 | 0.015 | **0.015** | 0.448 | 0.034 | 0.013 | **0.009** | 0.482 | 0.010 | 0.013 | 0.430 | 0.026 | **7.95x10^-4^** | 0.429 |
| rs1260326 | *GCKR* | 2 | 27584444 | typed | T | 0.201 | 0.026 | 0.016 | 0.101 | 0.478 | 0.021 | 0.014 | 0.144 | 0.394 | 0.020 | 0.013 | 0.132 | 0.022 | **0.008** | 0.919 |
| rs2097677 | *IL6* | 7 | 22699364 | imp | G | 0.778 | -0.013 | 0.015 | 0.388 | 0.863 | -0.034 | 0.019 | 0.071 | 0.895 | -0.045 | 0.021 | **0.033** | -0.027 | **0.009** | 0.656 |
| rs6734238 | *IL1F10* | 2 | 113557501 | imp | A | 0.362 | -0.017 | 0.013 | 0.194 | 0.083 | -0.039 | 0.023 | 0.080 | 0.110 | -0.038 | 0.021 | 0.074 | -0.026 | **0.009** | 0.510 |
| rs2847281 | *PTPN2* | 18 | 12811593 | imp | G | 0.219 | 0.011 | 0.015 | 0.458 | 0.124 | 0.027 | 0.019 | 0.168 | 0.121 | 0.034 | 0.020 | 0.091 | 0.022 | **0.036** | 0.864 |
| rs2836878 | *PSMG1* | 21 | 39387404 | typed | A | 0.775 | -0.024 | 0.015 | 0.108 | 0.835 | -0.021 | 0.019 | 0.267 | 0.861 | -0.012 | 0.019 | 0.538 | -0.020 | 0.052 | 0.980 |
| rs12239046 | *NLRP3* | 1 | 245668218 | imp | T | 0.420 | 0.029 | 0.013 | 0.025 | 0.381 | 0.009 | 0.013 | 0.523 | 0.357 | 0.006 | 0.013 | 0.629 | 0.015 | 0.052 | 0.387 |
| rs340029 | *RORA* | 15 | 58682257 | typed | T | 0.608 | -0.021 | 0.013 | 0.098 | 0.903 | -0.014 | 0.024 | 0.559 | 0.866 | -0.016 | 0.019 | 0.398 | -0.019 | 0.054 | 0.993 |
| rs10745954 | *ASCL1* | 12 | 102007224 | imp | G | 0.593 | 0.024 | 0.013 | 0.066 | 0.794 | 0.023 | 0.016 | 0.140 | 0.823 | -0.015 | 0.017 | 0.394 | 0.014 | 0.105 | 0.119 |
| rs10778213 |  | 12 | 102019281 | typed | T | 0.451 | -0.028 | 0.013 | **0.026** | 0.147 | -0.014 | 0.020 | 0.470 | 0.184 | 0.015 | 0.017 | 0.384 | -0.013 | 0.147 | **0.085** |
| rs4903031 | *RGS6* | 14 | 72088989 | typed | G | 0.327 | -0.014 | 0.013 | 0.286 | 0.156 | -0.043 | 0.020 | **0.028** | 0.249 | 0.014 | 0.015 | 0.343 | -0.010 | 0.258 | **0.046** |
| rs4705952 | *IRF1* | 5 | 131867518 | imp | G | 0.396 | -0.019 | 0.015 | 0.197 | 0.554 | -0.013 | 0.014 | 0.366 | 0.462 | 0.005 | 0.015 | 0.728 | -0.009 | 0.279 | 0.405 |
| rs13233571 | *BCL7B* | 7 | 72609167 | imp | T | 0.063 | -0.035 | 0.026 | 0.187 | 0.092 | 0.008 | 0.022 | 0.709 | 0.117 | -0.004 | 0.021 | 0.833 | -0.008 | 0.562 | 0.807 |
| rs12037222 | *PABPC4* | 1 | 39837548 | typed | NA | NA | NA | NA | NA | 0.884 | -0.005 | 0.020 | 0.801 | NA | NA | NA | NA | NA | NA | NA |
| rs9987289 | *PPP1R3B* | 8 | 9220768 | imp | G | 0.935 | -0.020 | 0.025 | 0.441 | NA | NA | NA | NA | 0.954 | -0.064 | 0.031 | **0.038** | NA | NA | NA |

Typed: SNP genotyped in at least 1 dataset; imputed: imputed SNP; TA: test allele, TAF: test allele frequency.

* Mean allele frequency from 3 SNP-chip data of SP2 cohort.

**Figure S1**: In the study, at least 6 readings of retinal arteriolar (in red) and venular (in blue) calibres were summarized as central retinal arteriolar (CRAE) and the central retinal venular (CRVE) equivalent, respectively from retinal fundus photograph using the Interactive Vessel Analysis Software (IVAN, University of Wisconsin). CRAE and CRVE were defined based on the revised Knudtson-Parr-Hubbard formula.

**
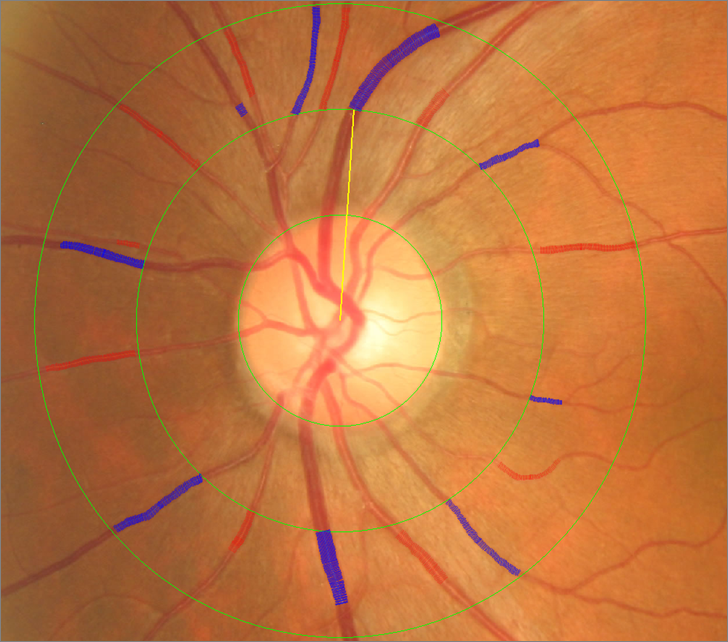
**

**Figure S2**: Forest plots comparing effect estimates from individual datasets and after fixed effect meta-analysis of all 14 SNPs that were observed to be significant for serum CRP association in the Singapore datasets (fixed-effect meta-p-value < 0.05, see table 2 of main text). A) rs207650 B) rs11265260 C) rs2794520 D) rs4420638 E) rs1183910 F) rs7310409 G) rs1169310 H) rs1892534 I) rs4537545 J) rs6901250 K) rs1260326 L) rs2097677 M) rs6734238 N) rs2847281


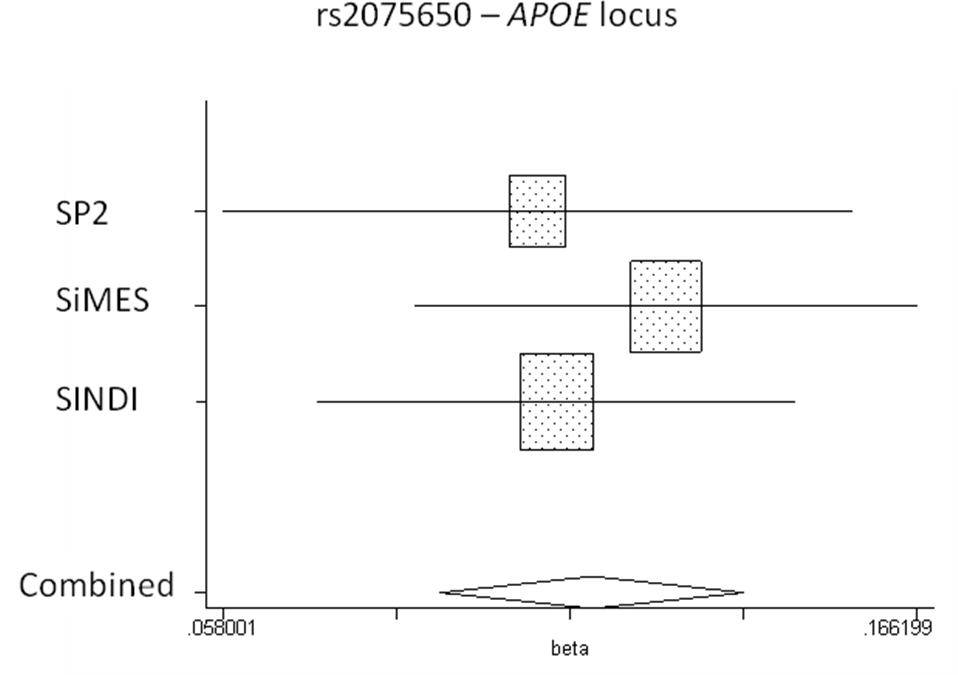

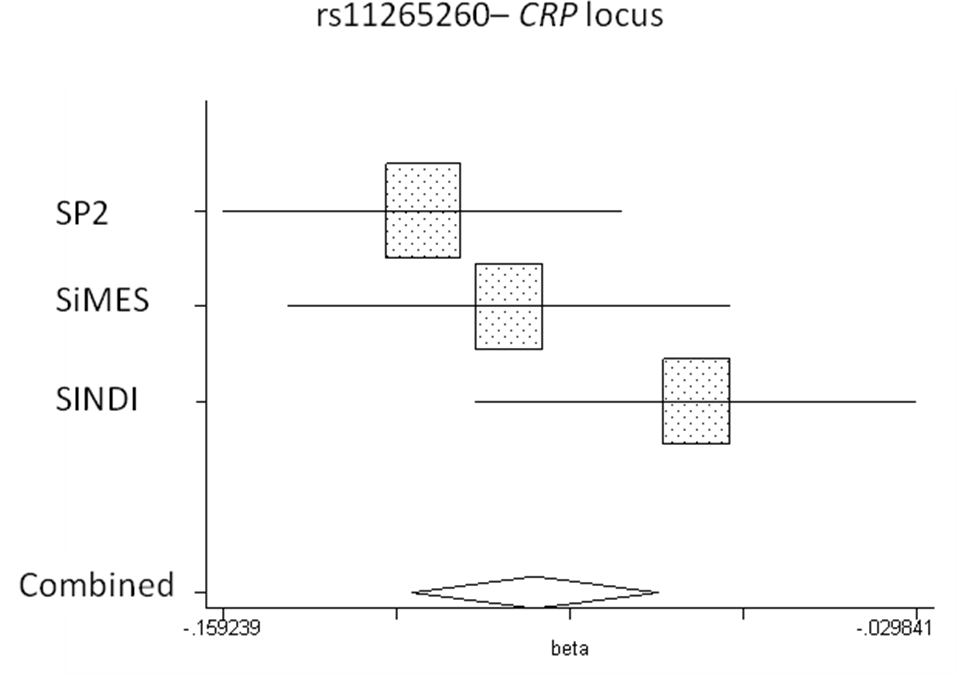

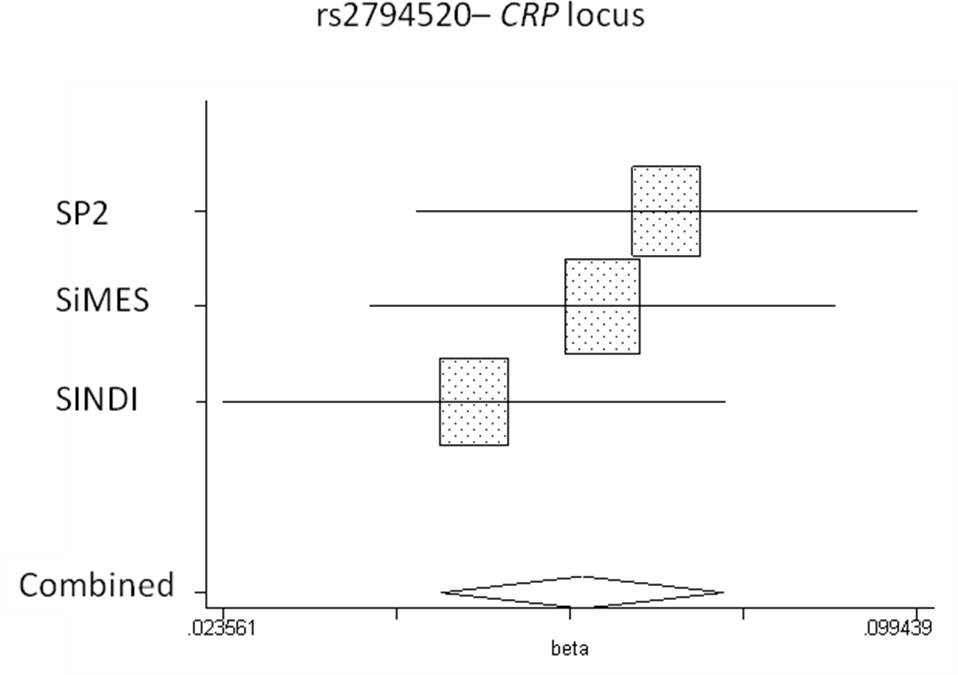


A)

C)

B)


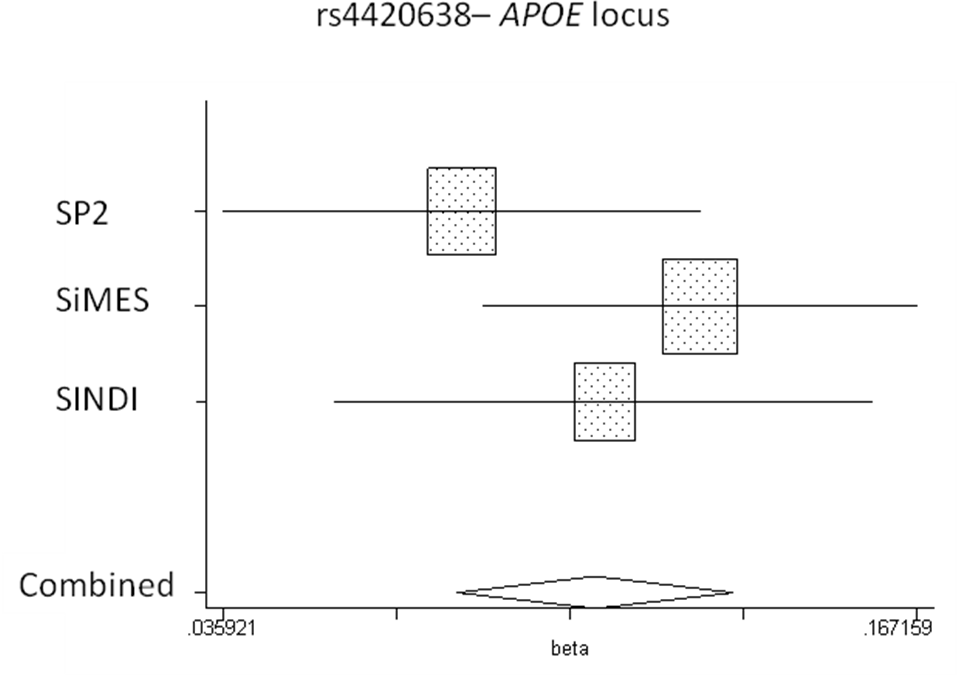

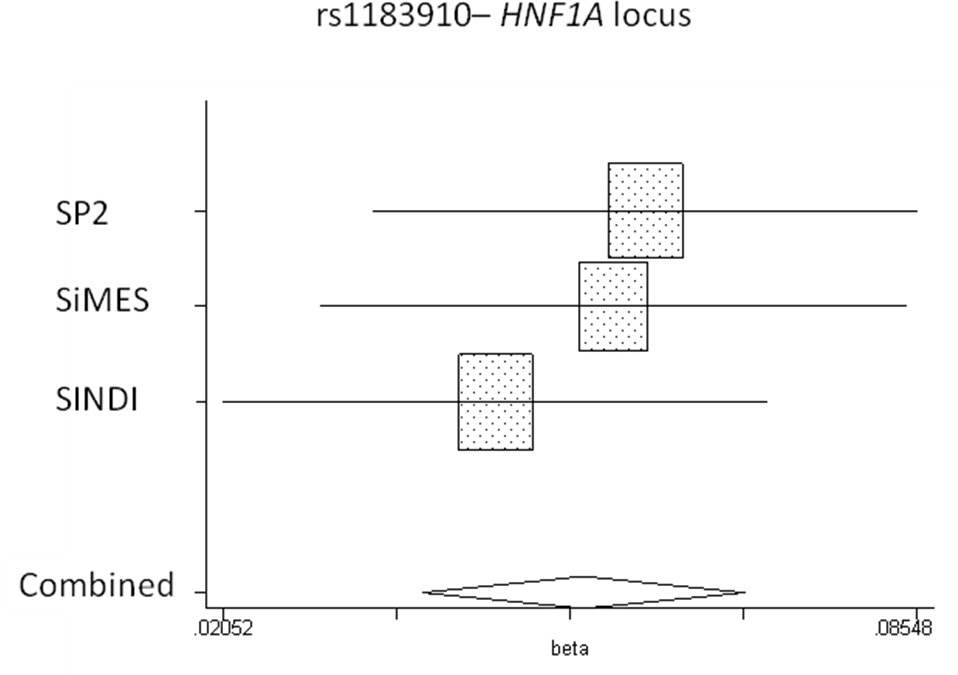

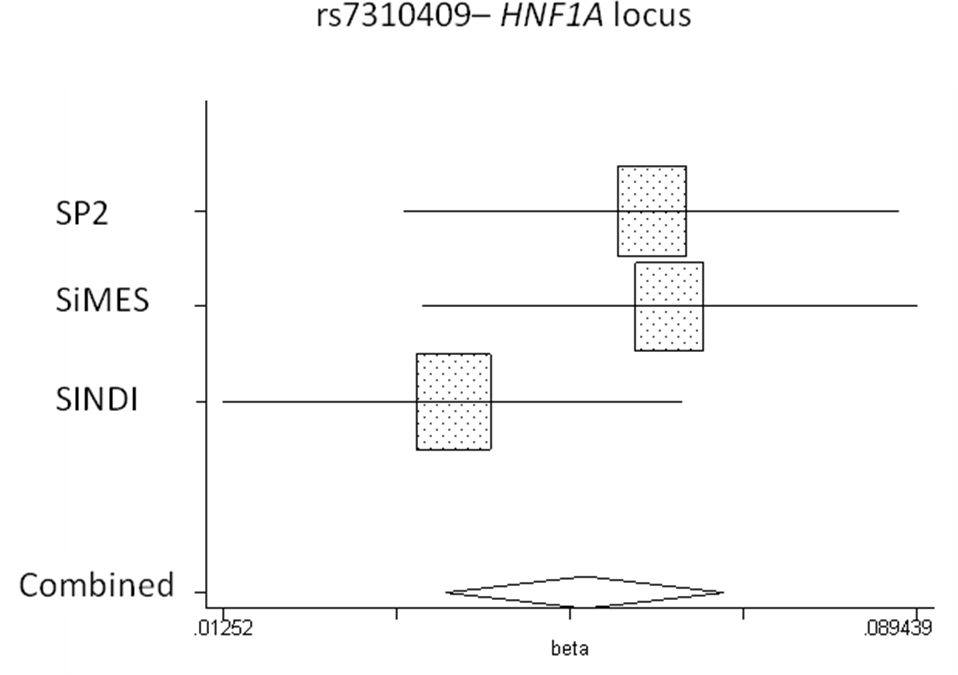

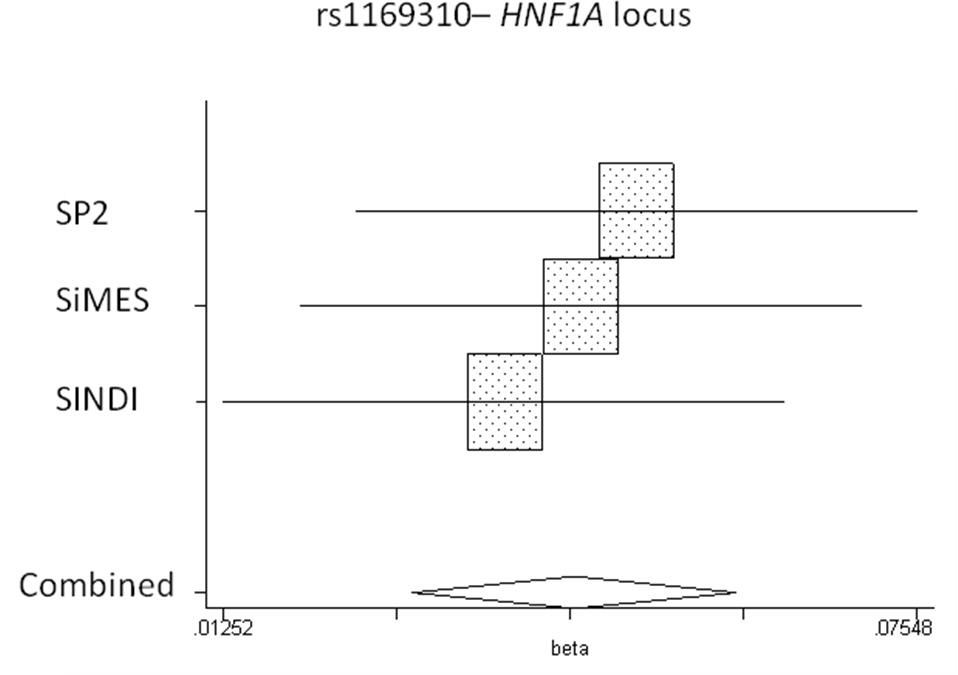

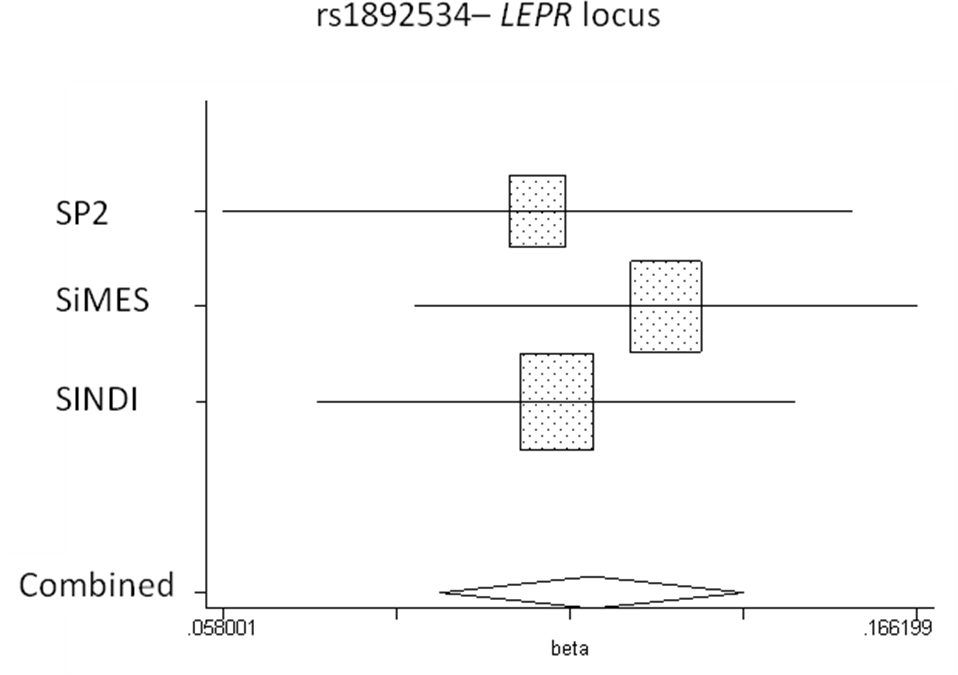

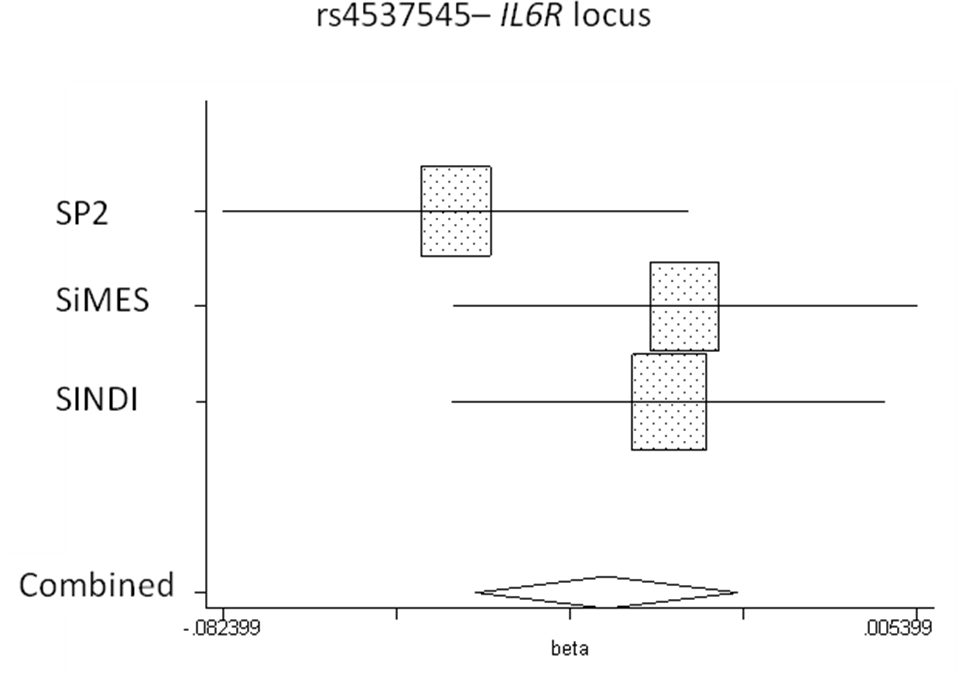

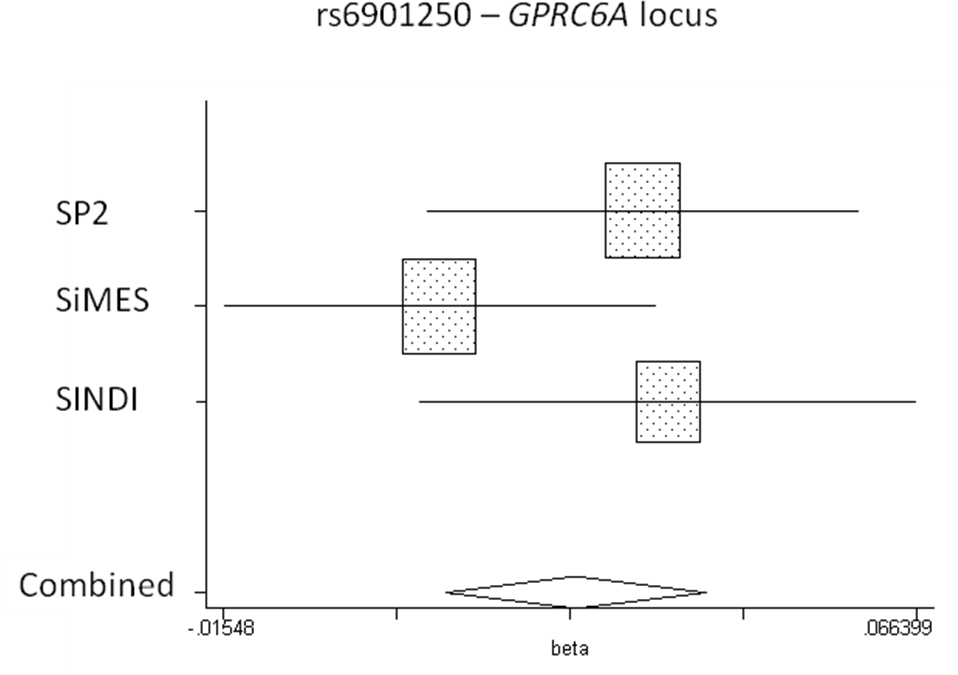

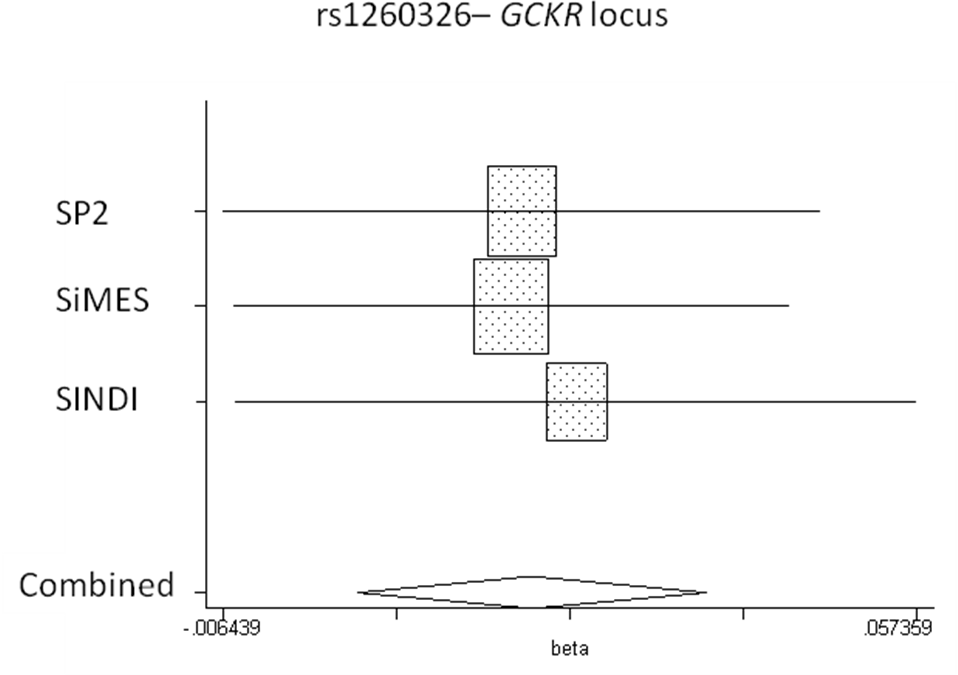

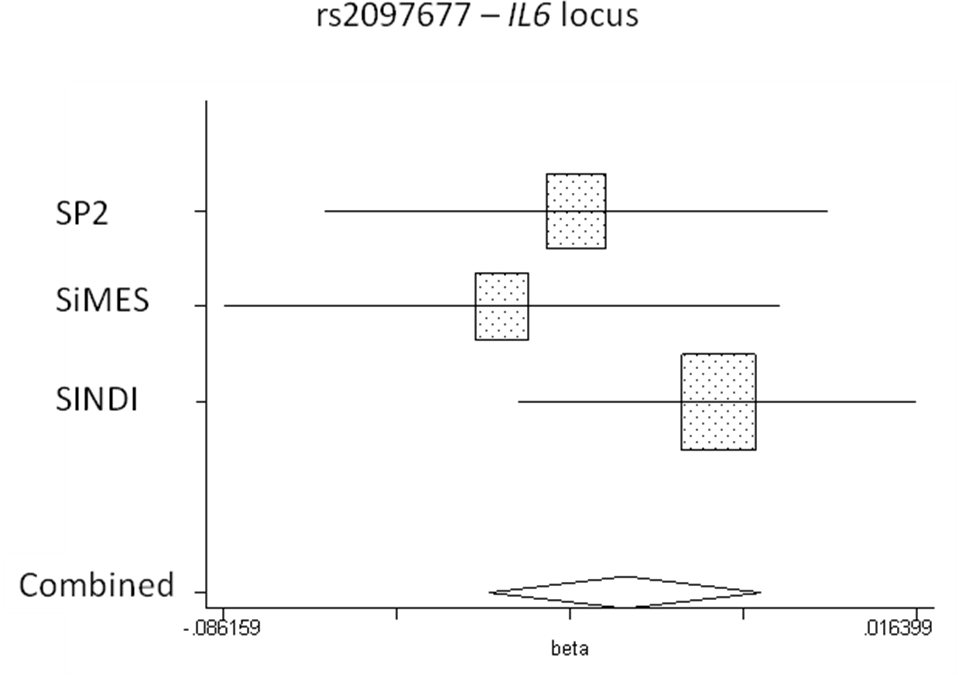

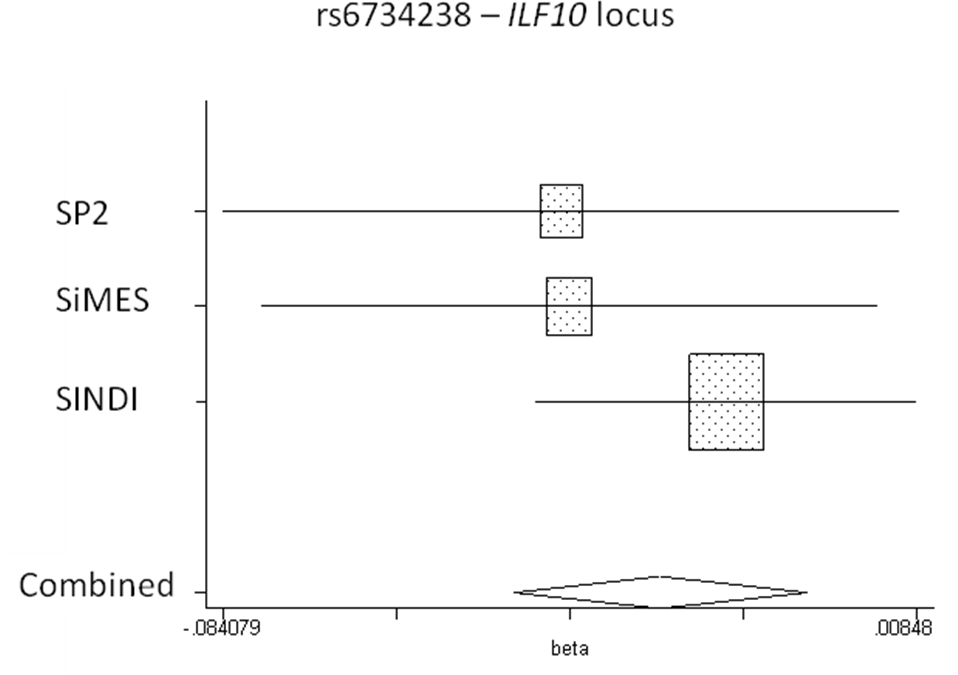

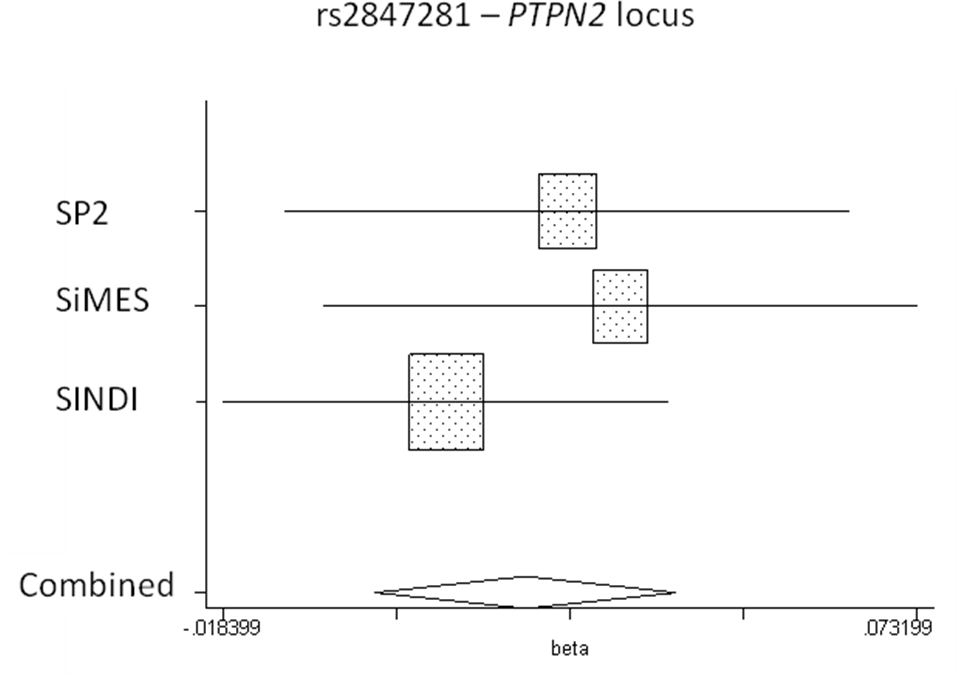


D)

E)

F)

G)

I)

H)

J)

K)

N)

L)

M)

**Figure S3**: Inter-population pair-wise varLD comparison of Han Chinese population (CHB) and European population (CEU) from HapMap ([www.hapmap.org](http://www.hapmap.org/)) and Singaporean Chinese (CHS), Singaporean Malays (MAS) and Singaporean Indian populations from SGVP ([www.statgen.nus.edu.sg/~SGVP/](http://www.statgen.nus.edu.sg/~SGVP/)) at A) 1Mb region surrounding the rs6901250 SNP at *GPRC6A* on chromosome 6 and B) 1Mb region surrounding the rs2847281 SNP at *PTPN2* on chromosome 18. Normalized scores calculated from population specific means and standard deviations. Positions of SNPs are indicated by red triangles.

**A)**


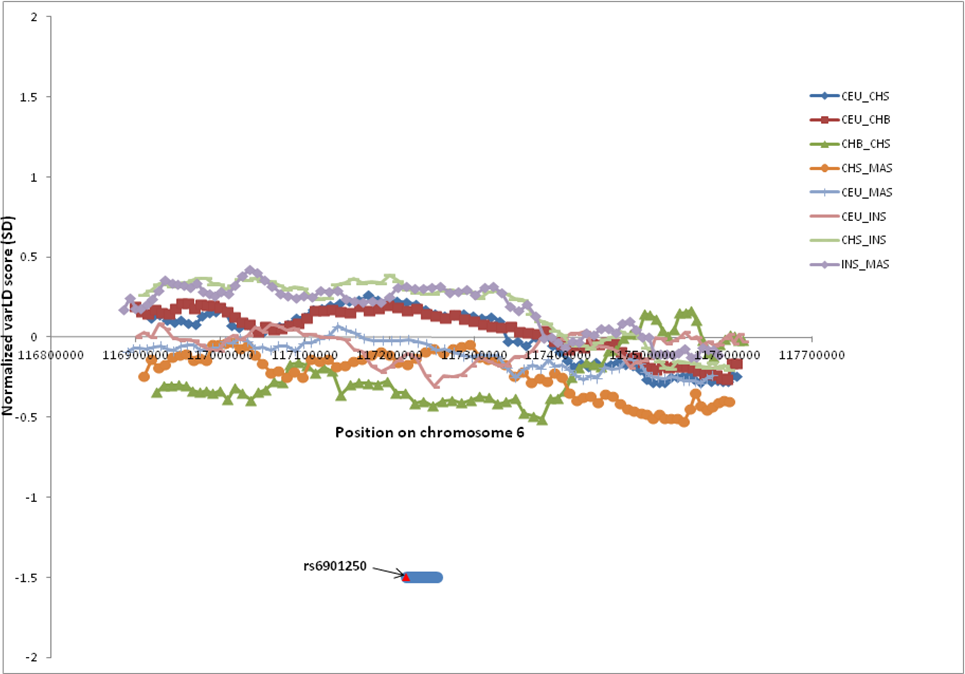


**B)**


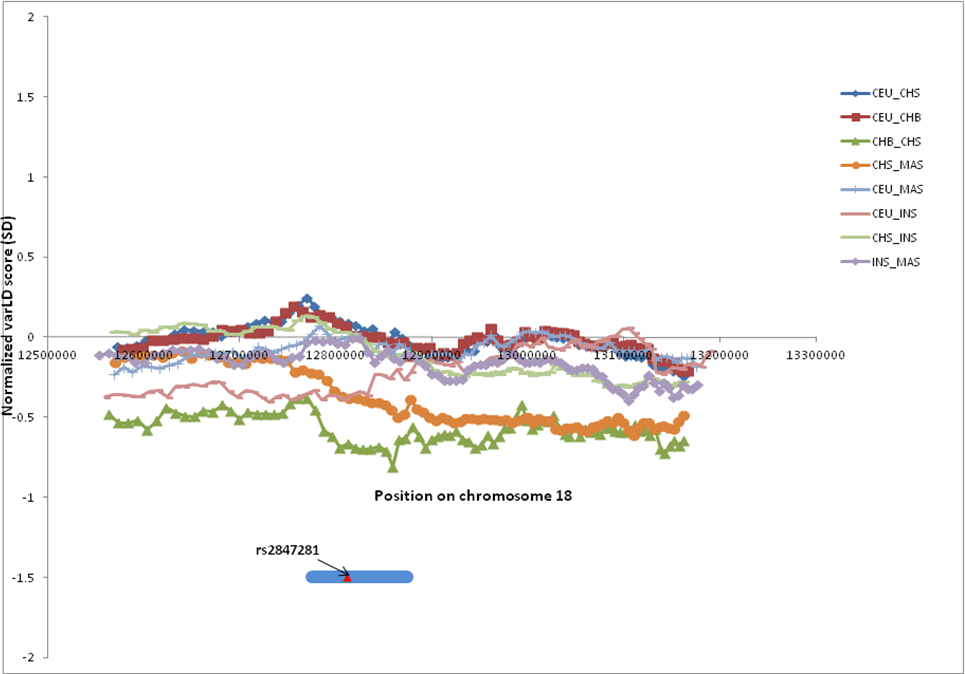


**Figure S4**: QQ-plot for CRP association p-values of fixed-effect meta-analysis of all 3 Singaporean datasets (SP2, SiMES and SINDI) (N=6,692) after removal of SNPs at 22 known CRP gene loci (100 kb upstream and downstream).


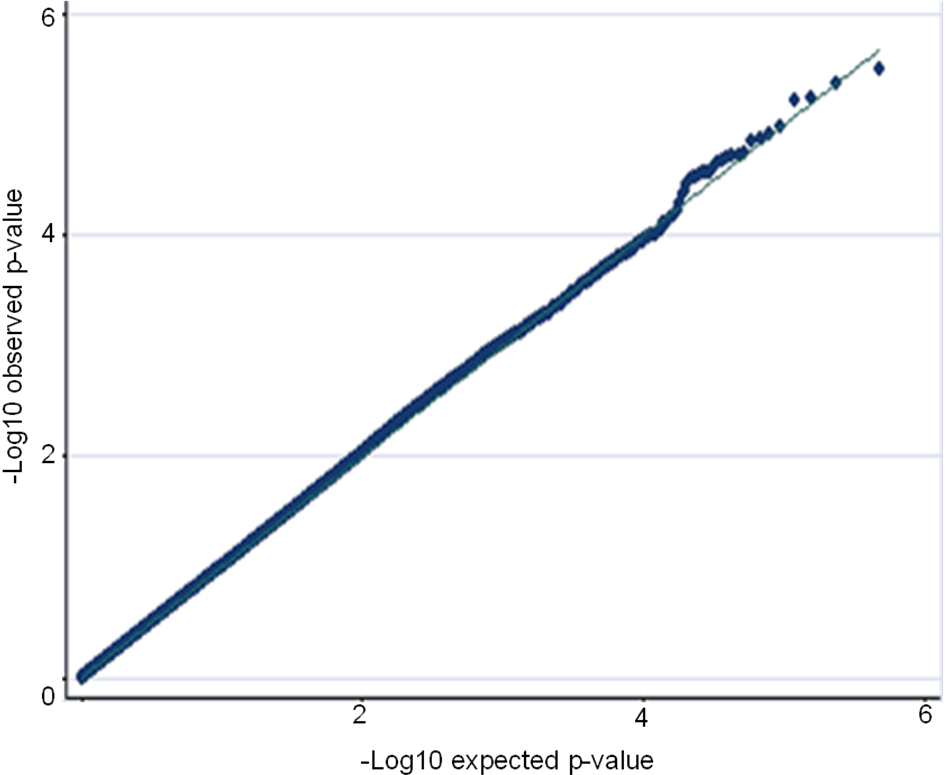

Supplement: File S1 — Combined supporting information file containing supporting tables and figure. File includes: Table S1, Sample QC threshold of GWAS datasets used in study; Table S2, SNP QC thresholds utilised for GWAS datasets used in study; Table S3, Details of 35 index CRP SNPs identified from previous GWAS; Table S4, Genome-wide hits for serum CRP association after meta-analysis of SINDI, SP2 and SiMES; Table S5, Full association results of known CRP index variants in SP2, SiMES and SINDI datasets and after meta-analysis; Figure S1, Central retinal arteriolar equivalent and the central retinal venular equivalent from retinal fundus photograph using the Interactive Vessel Analysis; Figure S2, Forest plots comparing effect estimates from individual datasets and after fixed effect meta-analysis of all 14 SNPs that were observed to be significant for serum CRP association in the Singapore datasets; Figure S3, Inter-population pair-wise varLD comparison of Han Chinese population (CHB) and European population (CEU) from HapMap and Singaporean Chinese (CHS), Singaporean Malays (MAS) and Singaporean Indian populations from SGVP at the GPRC6A and PTPN2 loci; Figure S4, QQ-plot for CRP association p-values of fixed-effect meta-analysis of all 3 Singaporean datasets (SP2, SiMES and SINDI) (N = 6,692) after removal of SNPs at 22 known CRP gene loci. (DOC) [file pone.0067650.s001.doc]
